# Leveraging piezo-augmented copper-induced bacterial death of sub-1 nm CuO-SrTiO_3-x_ heterojunction nanosheets for osteomyelitis eradication

**DOI:** 10.1186/s12951-026-04380-8

**Published:** 2026-04-15

**Authors:** Xueqing Wang, Kai Li, Wenyan Xu, Danyang Wang, Ying Wang, Junkun Feng, Yi Chen, Xiaoyi Liu, Zishan Xu, Xiaojia Liu, Shaohua Ge, Hong Liu, Jianhua Li

**Affiliations:** 1 Department of Biomaterials, School and Hospital of Stomatology, Shandong University & Shandong Key Laboratory of Oral Diseases & Shandong Engineering Research Center of Dental Materials and Oral Tissue Regeneration & Shandong Provincial Clinical Research Center for Oral Diseases, Cheeloo College of Medicine, Jinan, 250012 China; 2 State Key Laboratory of Crystal Materials, Shandong University, Jinan, 250100 Shandong China

**Keywords:** Heterojunction structure, Copper-induced metabolic toxicity, Piezocatalysis, Antibacterial, Osteomyelitis

## Abstract

Osteomyelitis is a refractory bacterial infection of bone, presenting significant clinical management challenges due to its limited therapeutic outcome. Here, we developed heterojunction-structured sub-1 nm copper oxide-decorated strontium titanate nanosheets (Cu-STO NSs), which integrate piezocatalytic reactive oxygen species (ROS) generation with augmented copper-induced bacterial death for synergistic osteomyelitis treatment. Under ultrasound (US) irradiation, the oxygen vacancies and heterojunction structure of Cu-STO NSs facilitate the separation of piezo-generated electron–hole pairs while reducing energy barriers, thereby enhancing ROS generation. This process is augmented by the US-accelerated Cu^2+^/Cu^+^ redox cycling, which enables sustained hydroxyl radicals supply via Fenton-like reactions. The US-driven piezocatalytic action simultaneously enhances bacterial membrane permeability, facilitating intracellular copper overload and thereby triggering copper-induced metabolic toxicity in *Staphylococcus aureus* (*S. aureus*). Subsequent release of bioactive Cu and Sr ions from Cu-STO NSs significantly upregulates osteogenic differentiation of bone marrow stromal cells. In rat osteomyelitis, Cu-STO NSs achieve efficient bacterial eradication while stimulating on-demand bone regeneration. This work thus presents a non-invasive, spatiotemporal controlled copper-induced bacterial death strategy that concurrently addresses antibacterial and osteogenic challenges in infected bone regeneration.

**Graphical Abstract:**

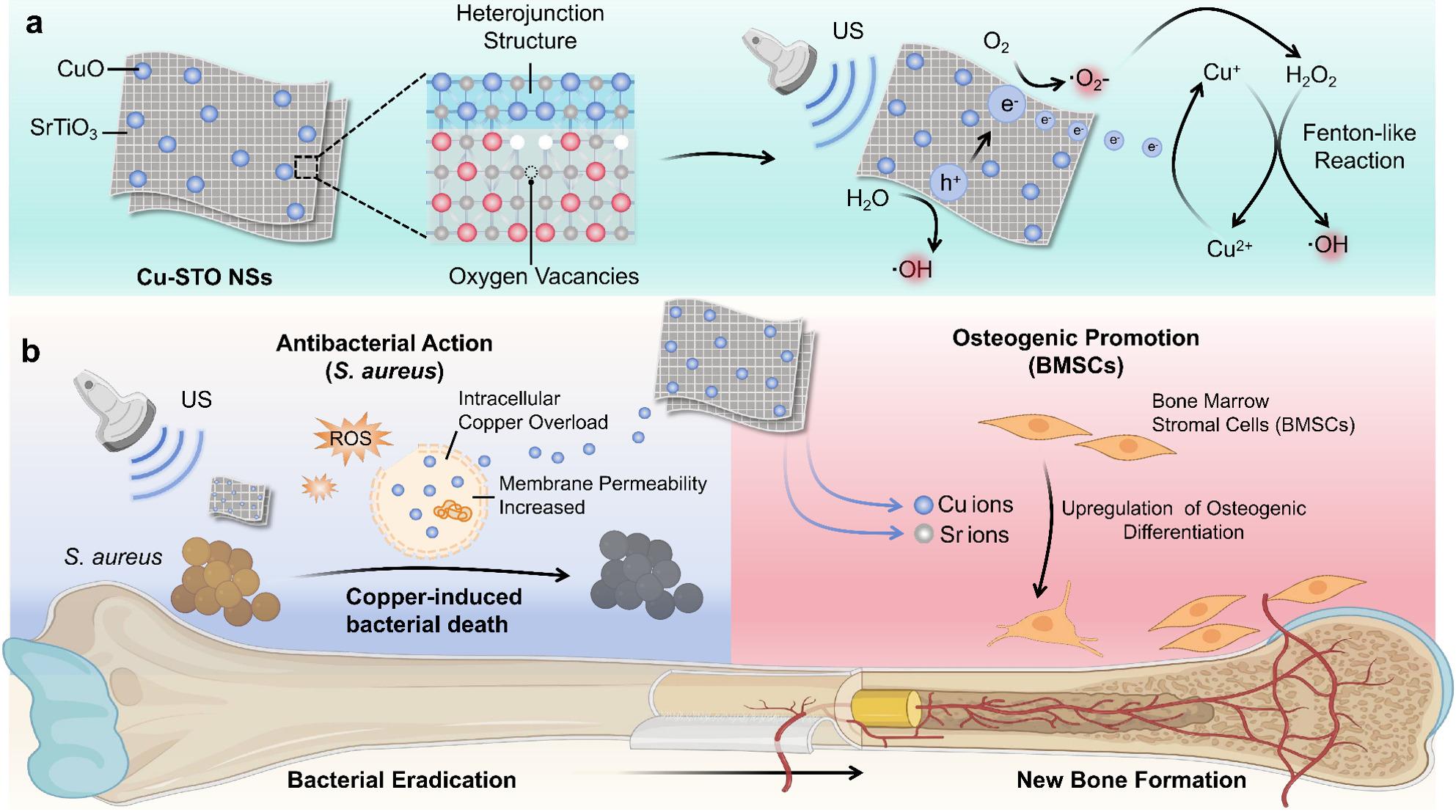

**Supplementary Information:**

The online version contains supplementary material available at 10.1186/s12951-026-04380-8.

## Introduction

Osteomyelitis, a devastating bone infection predominantly caused by bacteria such as *Staphylococcus aureus* (*S. aureus*, approximately 70% of cases), presents a persistent challenge in orthopedic practice [1, 2]. Conventional management through surgical debridement and prolonged antibiotic therapy is increasingly compromised by the rise of antimicrobial resistance and the immunosuppressive effects of certain antibiotics [3–5]. This therapeutic impasse has spurred the exploration of non-antibiotic strategies, including antimicrobial peptides [6, 7], metal ions [8, 9], and energy-based therapies like photo/sonodynamic treatment [10, 11]. Compared to traditional sonosensitizers which mainly depend on the cavitation effect, piezoelectric materials directly transduce ultrasound mechanical energy to drive chemical reactions for robust reactive oxygen species (ROS) generation [12, 13]. Among emerging approaches, cuproptosis—a copper-dependent cell death pathway—has shown considerable promise. This process involves excessive copper ions accumulation in the bacterial cells, which collectively disrupt metabolic homeostasis and induce oxidative stress, eventually leading to bacterial death [14]. However, the clinical translation of copper-induced bacterial death therapy faces a fundamental limitation: achieving bactericidal copper concentrations within bacterial cells without exceeding the cytotoxic threshold for mammalian cells [15]. The supplement of Cu ions should be strictly controlled between the minimal inhibitory concentration (~ 5 ppm) and the cytotoxicity concentration (~ 7.8 ppm) [16]. This narrow therapeutic window necessitates strategies to enhance copper-induced metabolic toxicity at low copper concentration in a controlled manner.

Recent evidence suggests that compromising bacterial membrane integrity can potentiate copper uptake [17], while metabolic modulation can increase bacterial vulnerability to copper toxicity [18]. Piezoelectric materials under mechanical stress generate built-in electric fields that drive charge separation and ROS generation, which can break down bacterial membrane integrity [19–21]. Ultrasound (US) offers deep tissue penetration and can precisely focus on the infected area, thus minimizing damage to surrounding healthy tissues and making it particularly suitable for treating bone infections [22–24]. This led us to hypothesize that piezoelectric catalysis could provide a platform for enhancing the efficacy of copper-induced bacterial death. However, copper ions are usually supplemented externally, how to integrate copper and piezocatalysis for controlled copper-induced bacterial death therapy remains a challenge.

**Scheme 1:**
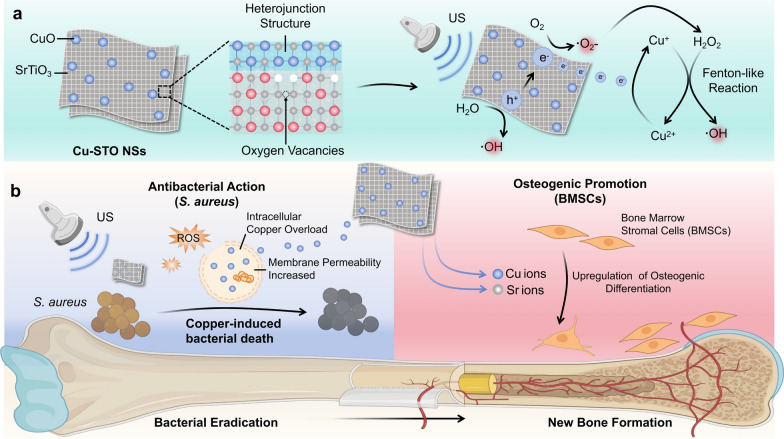
Mechanism of US-driven piezocatalytic therapy for osteomyelitis using Cu-STO sub-nanosheets. (**a**) Under US irradiation, the CuO/SrTiO_3_ heterojunction leverages piezoelectricity to boost ROS yield and sustain a catalytic Cu^2+^/Cu^+^ Fenton-like cycle for radical production. (**b**) At the infection site, the synergistic ROS attack and augmented copper overload disrupt bacterial metabolism, inducing bacterial copper-induced metabolic toxicity, while released Cu^2+^/Sr^2+^ ions synergistically promote osteogenesis and bone repair

Herein, we engineered sub-1 nm copper oxide/strontium titanate heterojunction nanosheets (Cu-STO NSs) for US‑triggered antibacterial therapy. Strontium titanate (STO) was selected as the piezoelectric base due to its (i) excellent biocompatibility and osteogenic potential via Sr^2^⁺ release [25, 26], (ii) ability to exhibit enhanced piezoelectricity at nanoscale thickness [27], (iii) defect‑tunable catalytic performance [28], and (iv) suitability for forming a heterojunction with CuO to synergize piezocatalysis and copper‑mediated antibacterial effects. The formation of Cu-STO NSs heterojunction structure not only achieves efficient Cu loading but also enhances piezocatalytic performance by improving charge separation while lowering energy barriers. Under US irradiation, this system establishes a self-sustaining Cu^2+^/Cu^+^ catalytic cycle that continuously supplies hydroxyl radicals via Fenton-like reactions. Concurrently, the US-driven piezocatalytic process increases bacterial membrane permeability, facilitating copper influx and disrupting cellular homeostasis, which collectively induce potent copper-induced metabolic toxicity in *S. aureus*. Additionally, Cu and Sr ions released from Cu-STO NSs promote osteogenic differentiation of bone marrow-derived mesenchymal stromal cells (BMSCs). In vivo experiments confirmed that Cu-STO NSs effectively achieve on-demand infection clearance and stimulate subsequent bone regeneration. Thus, this piezo-augmented copper-induced bacterial death strategy provides an US-controlled antibacterial therapy for osteomyelitis (Scheme 1).

## Experimental section

### Materials

Titanium hydride (TiH_2_), hydrogen peroxide (H_2_O_2_, 30%), strontium hydroxide octahydrate (Sr(OH)_2_·8H_2_O), copper(II) nitrate trihydrate (Cu(NO_3_)_2_·3H_2_O), sodium carbonate (Na_2_CO_3_), 2’,7’-Dichlorodihydrofluorescein diacetate (DCFH-DA), rhodamine B (RhB), acetone, sodium sulfate (Na_2_SO_4_), Neocuproine, Potassium phosphate monobasic (KH_2_PO_4_), Sodium hydroxide (NaOH), Ascorbic acid (VC), Bathocuproine disulfonate (BCS), 2.5% glutaraldehyde, crystal violet (CV) were purchased from Aladdin Biochemical Technology Co., Ltd. (Shanghai, China). STO nanoparticles (NPs) with a diameter of <100 nm were purchased from Beijing InnoChem Science & Technology Co., Ltd. Ammonium molybdate was procured from Macklin Biochemical Co., Ltd (Shanghai, China). O-nitrophenyl-β-D-galactopyranoside (ONPG), Bradford Protein Assay Kit, Cell counting kit-8 (CCK-8), Reactive Oxygen Species assay kit and Alkaline phosphatase (ALP) staining solution were obtained by Beyotime Biotechnology Co., Ltd. (Shanghai, China). Live/Dead BacLight Bacterial Viability Kit and Alizarin Red S (ARS) were purchased by Sigma-Aldrich (USA). Fetal bovine serum (FBS) and α-MEM medium were obtained from Thermo Fisher Scientific (Waltham, MA, USA). 4% paraformaldehyde was purchased from Solarbio (Beijing, China). All chemicals were of analytical grade and used without further purification.

### Sample preparation and characterization

Strontium titanate sub-nanosheets (STO NSs) were synthesized via a hydrothermal route. In a typical procedure, 0.2 g of TiH_2_ was reacted with 50 mL of H_2_O_2_ under stirring for 6 h to form a yellowish gel. The resulting gel was mixed with a certain proportion of Sr(OH)_2_·8H_2_O (Aladdin) and subjected to hydrothermal treatment at 300 ℃ for 5 h. After cooling to room temperature, the product was collected by centrifugation (8000 rpm, 5 min), washed repeatedly with ethanol, and dried at 80 ℃ to obtain STO NSs.

Copper oxide/strontium titanate sub-nanosheets (Cu-STO NSs) were subsequently prepared via an ultrasound-assisted impregnation method. A total of 100 mL of 0.1 M Cu(NO_3_)_2_·3H_2_O aqueous solution (Aladdin) was added to a suspension of STO NSs (0.4 g in 100 mL deionized water) under stirring. The pH was adjusted to 10.5 using 0.05 M Na_2_CO_3_ solution, followed by ultrasonication (40 kHz, 80 W) for 1 h. The resulting precipitate was collected by centrifugation, washed, dried at 80 ℃, and finally calcined at 300 ℃ for 2 h in air to obtain Cu-STO NSs.

For comparison, CuO NPs were synthesized following the same ultrasound-assisted impregnation procedure in the absence of STO NSs. STO-Annealing (STO-A) NSs were prepared by calcining STO NSs at 800 ℃ for 3 h in a muffle furnace under air. STO NPs (< 100 nm in diameter) were purchased from Beijing InnoChem Science & Technology Co., Ltd.

The morphology and microstructure of the samples were analyzed using high-resolution transmission electron microscopy (HRTEM, JEM-2100F, JEOL) operating at 200 kV, equipped with an energy-dispersive spectroscopy (EDS) detector. Atomic force microscopy (AFM, Dimension Icon, Bruker) was used to determine nanosheet thickness. The crystalline structure of the synthesized STO and Cu-STO NSs was characterized at room temperature with X-ray diffractometer (XRD, Smartlab3KW, Rigaku) using monochromatic Cu Kα radiation (λ = 1.5406 Å, 2θ = 25–80°). The surface chemical compositions of Cu-STO were detected by X-ray photoelectron spectroscopy (XPS, ESCALAB Xi + , Thermo Fisher Scientific). Electron spin resonance (ESR, EMX PLUS, Bruker) was employed to confirm oxygen vacancies (OVs). The piezoelectric response was measured by piezoresponse force microscopy (PFM, Dimension Icon, Bruker), and zeta potentials were obtained using a ZetaPALS analyzer (Bruker). To quantify the release of Cu and Sr ions, Cu-STO NSs were incubated in 0.9% NaCl at 37 ± 1 ℃ with agitation. At predetermined times, aliquots were taken, followed by centrifugation and analysis of the supernatants using ICP-OES (Agilent 5110).

### Measurement of piezocatalytic activity

Reactive oxygen species (ROS) generation was monitored using the fluorescent probe DCFH-DA. In a typical procedure, 10 mg of piezocatalyst was dispersed in 20 mL of DCFH-DA solution (10 μM) and stirred for 30 min in the dark. The suspension was then subjected to US irradiation (1 W cm^−2^, 50% duty cycle, 1 MHz) using an ultrasonic therapy instrument (WED-100, WELLD) at room temperature. 1 mL aliquots were collected after 1 and 5 min, centrifuged, and the fluorescence of the supernatant was measured using a fluorescence spectrometer (F-4700, Hitachi) at an excitation wavelength of 488 nm and an emission wavelength of 525 nm.

Piezocatalytic degradation of RhB was evaluated by dispersing 5 mg of catalyst in 5 mL of RhB (10 mg L^−1^). The mixture was kept in the dark for 1 h to establish adsorption–desorption equilibrium. After 5 min of ultrasonication (1 W cm^−2^, 1 MHz) in the dark, the mixture was centrifuged, and the absorption spectrum of RhB in the supernatant was recorded from 400 to 700 nm using a UV–Vis spectrophotometer (UV-3600, Shimadzu).

ESR spectroscopy (EMX PLUS, Bruker) was used to identify radical species. In the experiment, 5 mg of piezocatalyst were dispersed in 10 mL DI water. To detect •OH and •O_2_^−^, 1.5 mL of catalyst suspension was mixed with 0.2 mL of DMPO (1 M) or 0.3 mL of DMSO (1 M), respectively, and analyzed after 5 min of US irradiation (1 MHz, 1 W cm^−2^).

Electrochemical impedance spectroscopy (EIS) and piezoelectric current measurements were performed on an electrochemical workstation (CHI 660E, Chenhua). The working electrode was prepared by coating a mixture of piezocatalyst (10 mg), acetone (0.5 mL), and Nafion (20 μL, 5%) onto a glassy carbon electrode, followed by drying in air for 12 h. A platinum sheet and Ag/AgCl electrode served as counter and reference electrodes, respectively. EIS was recorded from 0.1 to 10^5^ Hz with an AC amplitude of 5 mV. Piezocurrent was measured under ultrasonication in 0.5 M Na_2_SO_4_ electrolyte.

H_2_O_2_ production was quantified by an iodide-based method [29]. Typically, 50 mg of catalyst was dispersed in 50 mL of 10 wt% ethanol and sonicated (1 MHz, 1 W cm^−2^). Every 5 min, 3 mL of suspension was centrifuged (12,000 rpm, 5 min). Then, 500 µL of supernatant was reacted with 2 mL of KI (0.1 M) and 50 µL of ammonium molybdate (0.01 M) for 10 min. The absorbance spectrum from 300 to 500 nm was recorded using a UV–Vis spectrophotometer (UV-3600, Shimadzu).

A neocuproine-based colorimetric assay was used to detect Cu^+^. Neocuproine (1.04 mg, Aladdin) was dissolved in 5 mL ethanol and diluted fivefold with ultrapure water. A pH 6.5 buffer was prepared using KH_2_PO_4_ and NaOH. Cu-STO dispersion (500 μg mL⁻^1^) was mixed with 0.75 mL buffer, 1.0 mL Cu-STO solution, and 0.8 mL neocuproine solution. The mixture was irradiated with US (1.5 W cm^−2^, 50% duty cycle, 1 MHz, 5 min) or left untreated. The absorbance spectrum from 350 to 700 nm was measured using a UV–Vis spectrophotometer (UV-3600, Shimadzu).

Ultraviolet–visible diffuse reflection spectra (UV–vis DRS) were obtained using a UV–vis spectrophotometer (UV-3600, Shimadzu) from 200 to 800 nm. The band gaps (E*g*) of STO and CuO were estimated from the absorption data using the Tauc plot method based on the following equation:

1 $$\it \left(ahv\right)^{2} = A \left(hv - E_{g}\right)$$

where A was absorbance;* a* was the absorption coefficient; *hv* was photon energy; E*g* was the band gap. The valence band (VB) positions were determined by XPS (ESCALAB Xi + , Thermo Fisher Scientific). The VB edge values relative to the normal hydrogen electrode (NHE) were calculated using:

2 $$E_{VB, NHE} = \varphi + E_{VB, XPS} - 4.44$$

where φ = 4.8 eV represents the work function of XPS spectrometer. The conduction band (CB) positions were subsequently derived from:

3 $$E_{CB} = E_{g} - E_{VB} $$

First-principles density functional theory (DFT) calculations were performed using the QUANTUM-ESPRESSO package. The exchange–correlation effects were described within the generalized gradient approximation of Perdew–Burke–Ernzerh of coupled with the projector-augmented wave method and ultrasoft pseudopotentials. A plane-wave energy cutoff of 500 eV and a 4 × 4 × 1 Monkhorst–Pack k-point mesh was employed for slab models. A vacuum layer exceeding 15 Å was introduced to prevent spurious periodic interactions. Structural models were relaxed until the energy and atomic forces converged to 1.0 × 10^–4^ eV and 0.05 eV/Å, respectively. The calculations incorporated van der Waals interactions via the DFT-D3 correction and applied a Hubbard U value of 4 eV to the Fe d-orbitals to account for strong electron correlations.

### Antibacterial assessment in vitro

*Staphylococcus aureus (S. aureus*, ATCC 12598) and methicillin-resistant *S. aureus* (MRSA, CCTCC AB 16465) were cultured in sterile LB medium. The antibacterial activity of STO and Cu-STO was evaluated using the spread plate method based on colony-forming units (CFU). Bacterial suspensions were adjusted to a concentration of 10^7^ CFU mL^−1^ and incubated with piezocatalyst (100 μg mL^−1^) in EP tubes for 1 h, followed by US irradiation (1.5 W cm^−2^, 50% duty cycle, 1 MHz) for 10 min. The bacterial suspension was subjected to three serial tenfold dilutions. Subsequently, 100 μL from the final dilution was spread onto an agar plate using a spreading rod and incubated for 14 h. Colonies were imaged and counted to quantify antibacterial efficacy.

To quantitatively delineate the contributions of ROS and copper ions to the antibacterial mechanism, control experiments were performed with specific inhibitors. *S. aureus* suspensions were adjusted to 10^7^ CFU mL^−1^ and allocated into the following treatment groups: (i) Control (no treatment), (ii) Control + BCS, (iii) Control + VC, (iv) Cu-STO (US+), (v) Cu-STO + BCS (US+), and (vi) Cu-STO + VC (US+). Corresponding agents were introduced at final concentrations of 100 μg mL^−1^ for Cu-STO, 50 μM for the copper chelator BCS, and 100 μM for the ROS scavenger VC. After a defined incubation period under standard conditions, bacterial viability was quantitatively assessed via the spread plate method. Colonies were imaged and enumerated to determine the CFU counts.

Bacterial viability was assessed using the Live/Dead BacLight Bacterial Viability Kit (Sigma-Aldrich 79,214) according to the manufacturer’s instructions. Fluorescence-stained bacteria were visualized using an epifluorescence microscope (BX-53, Olympus). For scanning electron microscopy (SEM), bacterial samples were washed with PBS, fixed with 2.5% glutaraldehyde for 1 h at 4 ℃, dehydrated in a graded ethanol series, and sputter-coated with gold before observation (Phenom Pro G5).

Membrane permeability was evaluated using ONPG (Beyotime ST429). *S. aureus* (10^8^ CFU mL^−1^) was pre-cultured in LB medium containing 10 μg mL^−1^ isopropyl β-D-1-thiogalactopyranoside for 24 h. After treatment with or without US (1.5 W cm^−2^, 50% duty cycle, 1 MHz, 10 min), 15 μL of bacterial suspension was mixed with 15 μL ONPG (12.5 mM), 15 μL dimethyl sulfoxide (7%), and 110 μL PBS. Absorbance at 420 nm was recorded using a microplate reader (SPECTROstar Nano, BMG LABTECH).

Protein leakage was quantified using a Bradford Protein Assay Kit (Beyotime P0006). Briefly, 1 × 10^8^ CFU mL^−1^ of *S. aureus* was incubated at 37 ℃ for 30 min after treatment with or without US (1.5 W cm^−2^, 50% duty cycle, 1 MHz, 10 min). The solution was then centrifuged at 10,000 rpm for 5 min at 4 ℃. The supernatant liquid was transferred to a 96-well plate and the protein leakage concentrations were measured by the Bradford Protein Assay Kit on a microplate reader and read at 595 nm.

Intracellular ROS of *S. aureus* cultured with different samples was examined by Reactive Oxygen Species assay kit (Beyotime Biotech, China). After employing the same method as described above for *S. aureus* incubation, the bacteria-adhered discs were washed with PBS twice. 500 μL PBS containing DCFH-DA (10 μM) was pipetted into each well for staining the bacteria for 30 min at 37 °C in darkness. The fluorescence-stained bacteria were examined using an epifluorescence microscope (BX-53, Olympus).

For transcriptomic analysis, bacterial suspension (100 μL) was mixed with 100 μL PBS (control) or 100 μL Cu-STO (100 μg mL^−1^), with or without US treatment (1.5 W cm^−2^, 50% duty cycle, 1 MHz, 10 min). After centrifugation (4000 rpm, 10 min, 4 ℃), bacterial pellets were frozen in liquid nitrogen. Total RNA extraction, sequencing, and bioinformatic analysis were performed by Qingdao OE Biotech Co., Ltd. Bioinformatic analyses were performed on the OECloud platform (https://cloud.oebiotech.com), including differential expression gene (DEG) screening, functional enrichment analysis (GO and KEGG), and visualization of results via integrated R‑based modules for generating volcano plots and heatmaps.

Biofilm inhibition was assessed by CV staining. *S. aureus* suspensions (10^6^ CFU mL^−1^) were incubated with piezocatalyst (100 μg mL^−1^) in 24-well plates and exposed to US (1.5 W cm^−2^, 50% duty cycle, 1 MHz) for 10 min with following incubation for 48 h. After staining with CV, excess dye was removed with PBS, and images were acquired. The stained biofilm was dissolved in acetic acid, and absorbance at 570 nm was measured to determine the anti-biofilm rate.

### In vitro cytocompatibility and osteogenic assessment

The mouse pre-osteoblast cell line MC3T3-E1 (RRID: CVCL_5437) were purchased from the Institute of Biochemistry and Cell Biology, Chinese Academy of Sciences, in Aug 2023. The cell line was confirmed to be free of mycoplasma contamination and was used within 10 passages. Bone marrow-derived mesenchymal stromal cells (BMSCs) were isolated from the femurs and tibiae of 3-week-old Sprague–Dawley rats as previously described [30]. All cells were maintained in α-MEM supplemented with 10% FBS, 100 μg mL^−1^ penicillin, and 100 μg mL^−1^ streptomycin at 37 °C in a humidified 5% CO_2_ atmosphere.

Cell viability was assessed through a CCK-8 assay according to the protocol of the manufacturer. MC3T3 cells were incubated with each sample for 24 h, and the absorbance at 450 nm was measured using a microplate reader (SPECTROstar Nano, BMG LABTECH).

ALP activity was assessed after 7 days of culture in osteogenic differentiation medium (containing 10 mM β-glycerophosphate, 300 μM VC, and 10 nM dexamethasone) with 100 μg mL^−1^ of samples. BMSCs were fixed with 4% paraformaldehyde and stained with ALP staining solution (Beyotime) for 30 min at room temperature. Stained cells were imaged under an optical microscope (BX-53, Olympus).

For ARS staining, BMSCs were cultured for 21 days in osteogenic medium containing 100 μg mL^−1^ of samples, fixed with 4% paraformaldehyde, and stained with 1% ARS (pH 8.0). Mineralized nodules were visualized microscopically, then dissolved in cetylpyridinium chloride, and absorbance was measured at 562 nm.

For immunofluorescence staining, BMSCs were seeded at 5 × 10^4^ cells/well in 24-well plates and cultured in osteogenic medium for 14 days. Cells were incubated with primary antibodies against BMP2 (1:200, Immunoway YM8215) and RUNX2 (1:200, Immunoway YM8347), followed by a goat anti-rabbit Alexa Fluor 488 secondary antibody (1:400, Abcam). Cytoskeleton was stained with FITC-phalloidin (Solarbio) and nuclei with DAPI. Images were acquired using an epifluorescence microscope (BX-53, Olympus).

Gene expression of ALP, RUNX2, and OPN was quantified by RT‑qPCR. BMSCs (1 × 10^4^ cells well^−1^) were seeded in 6-well plates and cultured in osteogenic medium with 100 μg mL^−1^ of samples. Total RNA was extracted using TRIzol after 14 days, reverse-transcribed using a Prime Script Kit, and amplified with SYBR Green on a CFX Connect Real-Time PCR System (Bio-Rad). The expression of osteogenic marker genes (ALP, RUNX2, and OPN) was normalized to GAPDH, and relative expression levels were calculated using the 2^–ΔΔCt^ method. The sequences of all primers used are provided in Table S1.

### Animal experiment

All animal procedures were approved by the Medical Ethical Committee of School of Stomatology, Shandong University (Protocol Number: 20250804). Male Sprague–Dawley rats (6 weeks old, ~ 200 g) were anesthetized via intraperitoneal injection of 2.5 wt% pentobarbital sodium. The dorsal fur was shaved and disinfected with 10% povidone-iodine. Rats were divided into three groups: control (physiological saline), Cu-STO (US–), and Cu-STO (US +). A bone defect (∼1.4 mm diameter) was created at the distal femoral plateau, and 100 μL of *S. aureus* suspension (10^8^ CFU mL^−1^) was injected into the marrow cavity to establish an osteomyelitis model. Physiological saline (300 μL) or Cu-STO NSs (0.1 mg mL^−1^, 300 μL) was injected into the marrow cavity, and the defect was sealed with bone wax. The Cu-STO (US +) group received US irradiation (1.5 W cm^−2^, 50% duty cycle, 1 MHz, 10 min) for 3 consecutive days.

On day 3 post-surgery, blood samples were collected for complete blood count analysis (BC‑2800vet). Femoral samples were harvested aseptically, and the bone marrow lavage fluid was plated on nutrient agar and incubated at 37 ℃ for 18 h to quantify the bacterial load. Additionally, femoral tissues were fixed in 4% paraformaldehyde for 48 h, decalcified in 10% EDTA‑2Na at 4 ℃ for 4 weeks, dehydrated, embedded in paraffin, and sectioned for hematoxylin and eosin (H&E) and Giemsa staining.

At 4 weeks post-surgery, femoral samples were fixed and scanned using a micro‑CT system (Skyscan 1176, Bruker). Three‑dimensional reconstructions were generated with CT‑Vox software, and bone morphological parameters including bone volume/total volume (BV/TV) and trabecular thickness (Tb. Th) were quantified using CT Analyzer software. Subsequently, the samples were decalcified, embedded, and sectioned for Masson’s trichrome staining and immunofluorescence staining of BMP‑2 (1:200, Immunoway YM8215) and RUNX2 (1:200, Immunoway YM8347).

### Statistical analysis

All data were expressed as mean values ± SD. A one-way ANOVA and Student’s t test were employed for significance analysis. *p < 0.05, **p < 0.01, ***p < 0.001 and ****p < 0.0001 were considered statistically significant.

## Results and discussion

### Synthesis and characterization of Cu-STO NSs

**Fig. 1:**
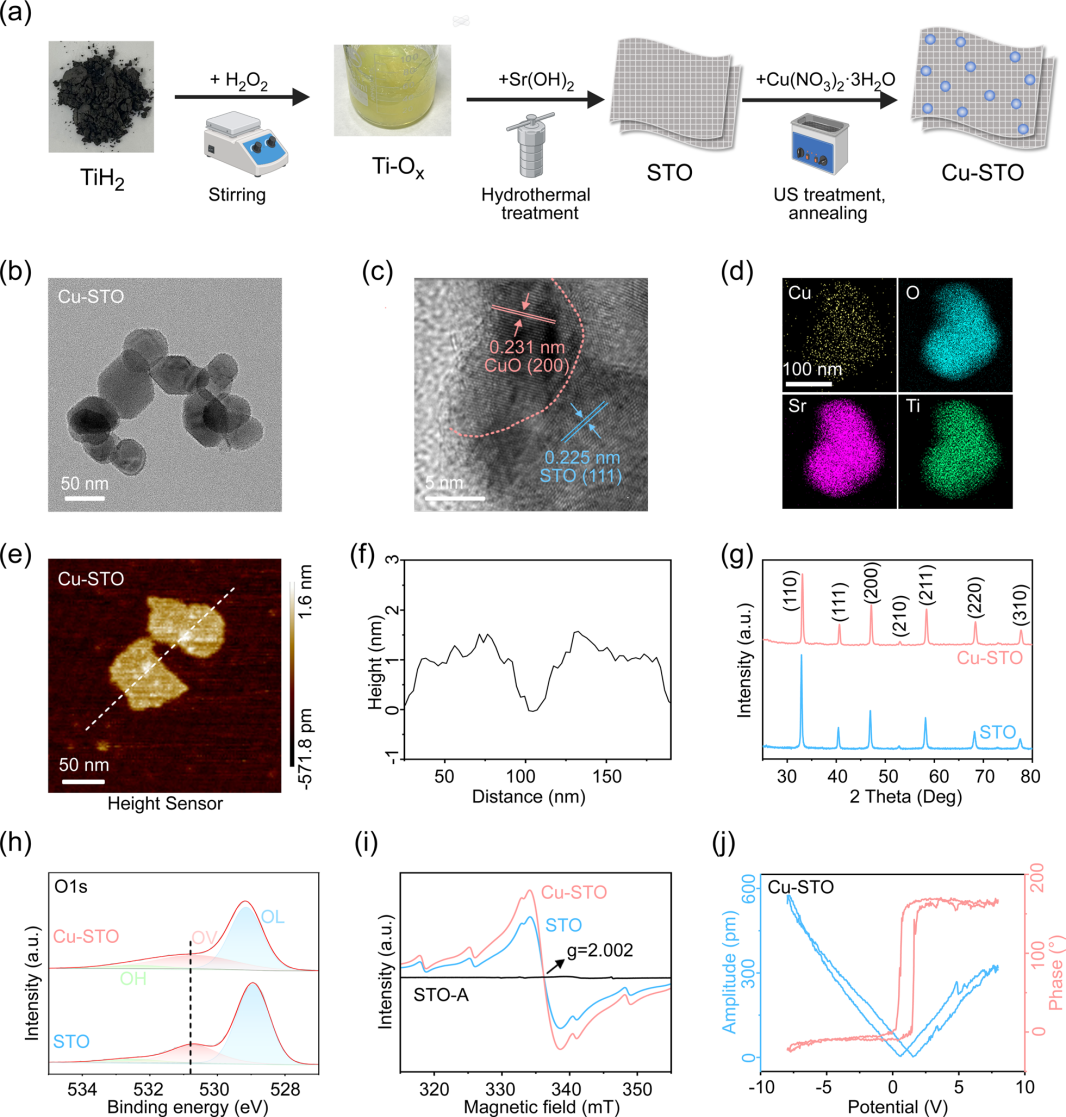
Synthesis and characterization of Cu-STO NSs. (**a**) Schematic diagram of the Cu-STO NSs fabrication process. (**b-d**) HRTEM image and EDX mapping images of Cu-STO NSs: (**b**) bright-field image, (**c**) magnified bright-field image, and (**d**) corresponding EDX mapping images. (**e**) AFM topography and (**f**) corresponding height profiles of Cu-STO NSs. (**g**) XRD patterns of STO and Cu-STO NSs. (**h**) High-resolution O 1 s XPS spectra of STO and Cu-STO NSs. (**i**) ESR spectra comparing the OVs concentrations in STO, STO-A and Cu-STO NSs. (**j**) Representative piezoresponsive phase-amplitude curve of Cu-STO NSs

The synthesis of Cu-STO NSs is schematically illustrated in Fig. 1a. Initially, TiH_2_ powder was oxidized with H_2_O_2_ solution, yielding a yellowish-green gel composed of titanium oxides (TiO*x*) and titanium oxohydrides (Ti(OH)*x*) [31]. Subsequently, STO NSs were synthesized via a hydrothermal reaction involving Sr^2+^, OH^−^, and the TiO*x*/Ti(OH)*x* gel. Finally, Cu-STO NSs were fabricated by depositing CuO onto the STO NSs using an US-driven impregnation method. Morphological characterization by high-resolution transmission electron microscopy (HRTEM) (Fig. 1b) showed that Cu-STO composites retain nanosheet morphology with lateral dimensions of 30–100 nm, consistent with pristine STO NSs (Fig. S1). Magnified bright-field image resolved characteristic lattice spacings of 0.231 nm and 0.225 nm, corresponding to CuO (200) and STO (111) planes, respectively (Fig. 1c). The elemental mapping via energy-dispersive X-ray spectroscopy (EDS) verified homogeneous distribution of Sr, Ti, O, and Cu throughout the nanosheets (Fig. 1d). Zeta potential measurements (Fig. S2) revealed a negative potential (-8.79 mV) for STO, and the intermediate potential (+ 2.94 mV) of Cu-STO confirms its successful composite formation via electrostatic interactions, further confirming Cu-STO heterojunction formation.

Moreover, atomic force microscopy (AFM) was employed to measure the thickness of the Cu-STO NSs (Fig. 1e). The 2D surface profile obtained from AFM measurements indicated that the thickness of Cu-STO NSs was approximately 1 nm (Fig. 1f), demonstrating the successful synthesis of sub-1 nm-scale nanosheet structures. X-ray diffraction (XRD) analysis (Fig. 1g) confirmed that the STO pattern exhibited characteristic perovskite peaks, while Cu-STO maintained near-identical diffraction profiles, indicating preserved host crystallinity after copper incorporation. X-ray photoelectron spectroscopy (XPS) spectra detected Sr, Ti, and O in STO, while Cu-STO additionally showed distinct Cu signatures (Fig. S3). Deconvolution of the O 1 s spectra (Fig. 1h) identified three constituent peaks for both materials. The intensified peak at 532.5 eV corresponds to surface hydroxyl groups, indicating oxygen vacancies (OVs) formation. The presence of OVs was further verified by electron spin resonance (ESR) spectroscopy (Fig. 1i), where both STO and Cu-STO exhibited a characteristic symmetric signal at g = 2.002. After annealing treatment, the STO-Annealing NSs (STO-A NSs) show a nearly flat curve, confirming the disappearance of OVs. The presence of OVs may enhance catalytic activity in piezoelectric materials by modulating electronic structures [32–35]. Piezoresponse force microscopy (PFM) with conductive probe directly quantified piezoelectric properties of STO and Cu-STO. Representative ferroelectric hysteresis loops and butterfly amplitude curves (Fig. 1j and S4a) were observed alongside voltage-dependent amplitude imaging (-10 to + 10 V), confirming pronounced piezoelectric behavior in both STO NSs and Cu-STO NSs. STO NSs has superior piezoelectric performance compared with both STO-A NSs and STO nanoparticles (NPs) (Fig. S4b, c), underscoring the important role of OVs and the ultrathin 2D architecture. As reported, oxygen vacancies can enhance local piezoelectric properties [36]. Moreover, a reduction in STO thickness significantly enhances remnant polarization [27], and the ultrathin 2D architecture is particularly advantageous for piezoelectric applications, as such materials undergo substantial deformation under mechanical stress to generate strong piezoresponses during ultrasonication [37]. Critically, the reduced carrier migration distance in this structure facilitates rapid transport of internally generated charges to the surface, substantially enhancing catalytic efficiency.

### Piezocatalytic activities of Cu-STO NSs

**Fig. 2:**
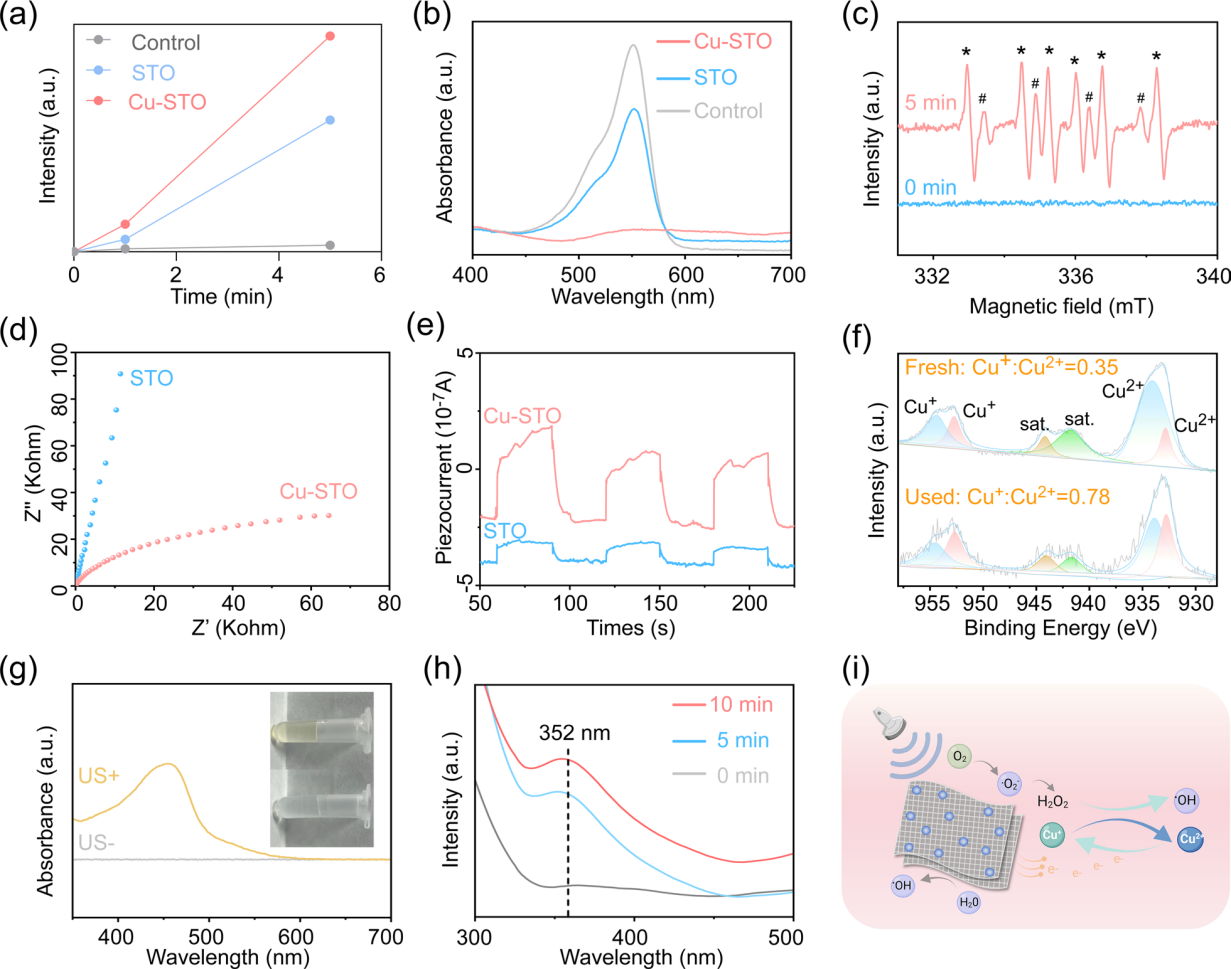
Piezocatalytic activities of Cu-STO NSs. (**a**) Time-dependent fluorescence intensity of DCFH indicating ROS generation under US. (**b**) UV–Vis absorbance spectra of RhB degradation under US stimulation. (**c**) EPR spectra for detecting •OH (labeled as #) and •O_2_- (labeled as *). (**d**) Nyquist plot and (**e**) piezoelectric current response curves of STO and Cu-STO under cycled on/off US stimulation. (**f**) High-resolution Cu 2p XPS spectra of Cu-STO before and after US exposure. (**g**) Neocuproine-based detection of Cu^+^. UV–vis spectra and solution color (inset) show the formation of yellow [Cu(neocuproine)_2_]^+^ (λmax = 452 nm) upon US activation of Cu-STO. (**h**) Time-dependent UV–vis spectra for H_2_O_2_ production. (**i**) Proposed mechanism of the self-sustaining catalytic cycle for continuous ROS generation via US-driven Fenton-like reactions

To quantify piezocatalytic efficacy, we assessed the ROS generation ability of Cu-STO NSs under US irradiation. Time-dependent ROS production monitored by DCFH-DA probe revealed significantly enhanced fluorescence intensity in Cu-STO NSs versus pristine STO (Fig. 2a). Piezocatalytic degradation of the model organic dye Rhodamine B (RhB) under US stimulation (5 min) exhibited the highest degradation efficiency in Cu-STO NSs (Fig. 2b). Radical speciation analysis via ESR spectroscopy with 5,5-dimethyl-1-pyrroline N-oxide (DMPO) spin traps identified •O_2_⁻ and •OH signals exclusively during US activation (Fig. 2c), mechanistically confirming ROS generation pathways.

Efficient charge transfer in piezocatalysts critically determines redox reaction efficacy. We thus performed electrochemical impedance spectroscopy (EIS) to evaluate charge migration capabilities. The Nyquist plot (Fig. 2d) revealed significantly reduced charge transfer resistance in Cu-STO versus pristine STO. This was corroborated by enhanced piezoelectric currents in Cu-STO under cyclic US vibration on ITO electrodes (Fig. 2e), confirming superior charge separation/transport that facilitates piezocatalytic reactions. Besides, XPS spectra of Cu-STO NSs before and after US stimulation were carried out (Fig. 2f). Deconvolution of Cu 2p3/2 spectra showed characteristic peaks at ~ 935 eV (Cu^2+^) and ~ 932 eV (Cu^+^). Notably, the integral area ratio of Cu^+^ to Cu^2+^ was significantly enhanced from 0.35 for fresh Cu-STO NSs to 0.78 for Cu-STO NSs with US stimulation, evidencing partial Cu^2+^ → Cu^+^ reduction on Cu-STO NSs. Neocuproine chromogenic assays further verified piezocatalytic-driven Cu^+^ generation (Fig. 2g). Concurrently, KI-based quantification demonstrated time-dependent H_2_O_2_ accumulation (352 nm monitoring; Fig. 2h). These findings establish a self-sustaining catalytic cycle: piezoelectric-driven Cu^2+^ reduction generates Cu^+^, which activates H_2_O_2_ to produce •OH via Fenton-like reactions (Fig. 2i), enabling continuous piezocatalytic-chemodynamic reactions.

### Mechanism study of piezocatalytic Cu-STO NSs

**Fig. 3:**
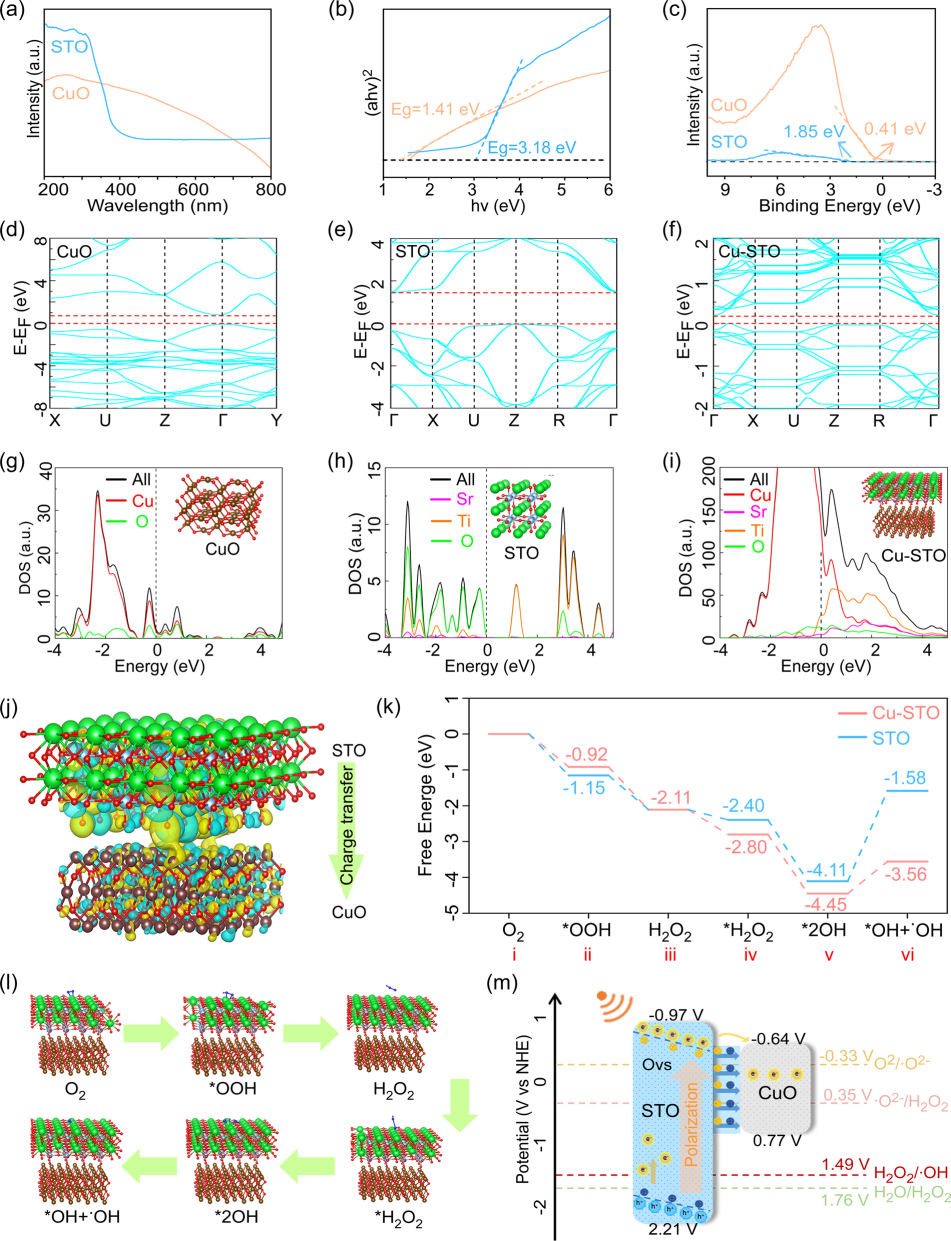
Mechanism of enhanced piezocatalytic performance in Cu-STO NSs. (**a**) UV–Vis DRS spectra and (**b**) Tauc plots for bandgap estimation of STO and CuO. (**c**) VB XPS spectra of STO and CuO. (**d-f**) Calculated band structures and (**g–i**) DOS for CuO, STO and Cu-STO. (**j**) Charge density difference at the Cu-STO heterojunction interface (yellow: electron accumulation; blue: electron depletion). (**k**) Energy diagrams and (l) key intermediate conformations of STO and Cu-STO in tandem reactions for •O_2_- to •OH conversion. (**m**) The proposed charge transfer mechanism across the heterojunction under US

To elucidate the mechanistic basis of US-driven piezocatalysis in Cu-STO heterojunction, we analyzed the band structure using ultraviolet–visible diffuse reflection spectroscopy (UV–Vis DRS). As shown in Fig. 3a, CuO exhibits stronger light absorption compared to STO. The corresponding bandgap (E*g*) values were calculated to be 1.41 eV for CuO and 3.18 eV for STO (Fig. 3b). XPS was used to determine the valence band (VB) positions, yielding binding energies of 0.41 eV for CuO and 1.85 eV for STO (Fig. 3c). After calibration to the normal hydrogen electrode (NHE) scale, the VB positions were 0.77 eV for CuO and 2.21 eV for STO, with corresponding conduction band (CB) positions at –0.64 eV and –0.97 eV, respectively.

The band structure of the Cu-STO NSs were further investigated using density functional theory (DFT) calculations. As shown in Fig. 3d-f, Cu-STO exhibits a narrowed bandgap relative to pristine STO due to CuO incorporation, consistent with UV–Vis experimental trends (1.41 eV *vs* 3.18 eV). This reduced bandgap facilitates enhanced electron–hole pair generation under US excitation. The density of states (DOS) results (Fig. 3g–i) reveal that STO exhibits sharp peaks, indicative of flat bands with strong localization, high effective electron mass, and limited mobility. In contrast, Cu-STO shows broader energy bands, suggesting enhanced electron delocalization, reduced effective mass, and higher carrier mobility. Charge density difference mapping visualized significant electron accumulation on CuO domains and depletion on STO regions, confirming interfacial electron transfer across the heterojunction (Fig. 3j). Oxygen vacancy-incorporated models demonstrated enhanced catalytic kinetics for •O_2_- to •OH conversion. Free energy diagrams (Fig. 3k) revealed reduced energy barriers on Cu-STO versus pristine STO, particularly for: HOO* dissociation to O* + •OH (ΔΔG‡ = -0.40 eV) and O* protonation to HO* (ΔΔG‡ = -0.34 eV). Key intermediate configurations (Fig. 3l and S5) corroborate accelerated •OH desorption and proton consumption kinetics.

Based on these findings, a schematic band alignment diagram for the Cu-STO heterojunction was constructed (Fig. 3m). This configuration facilitates electron transfer from CB of STO to CuO domains, consistent with the charge density distribution analysis. The resulting spatial charge separation efficiently drives US-activated oxygen reduction and water oxidation reactions, thereby enhancing ROS generation and catalytic performance.

### In vitro antibacterial performance of Cu-STO NSs

To determine the concentration range of the as-prepared samples that effectively combats bacteria while remaining non-harmful to osteoblasts, we systematically investigated the cellular activity of mouse pre-osteoblasts (MC3T3-E1) after co-culture with varying concentrations of Cu-STO NSs. As shown in Fig. S6, the impact of samples on cell viability exhibited a distinct concentration-dependent manner. When Cu-STO NSs’ concentration was below 100 µg mL^−1^, the relative survival rate of MC3T3-E1 cells showed no significant difference (p > 0.05) compared to the blank control group, remaining above 85%. This result indicates that Cu-STO NSs exhibit no significant toxicity and demonstrate excellent cytocompatibility towards osteoblasts within this concentration range. Consequently, a concentration of 100 µg mL^−1^ was selected for all subsequent experiments.

**Fig. 4:**
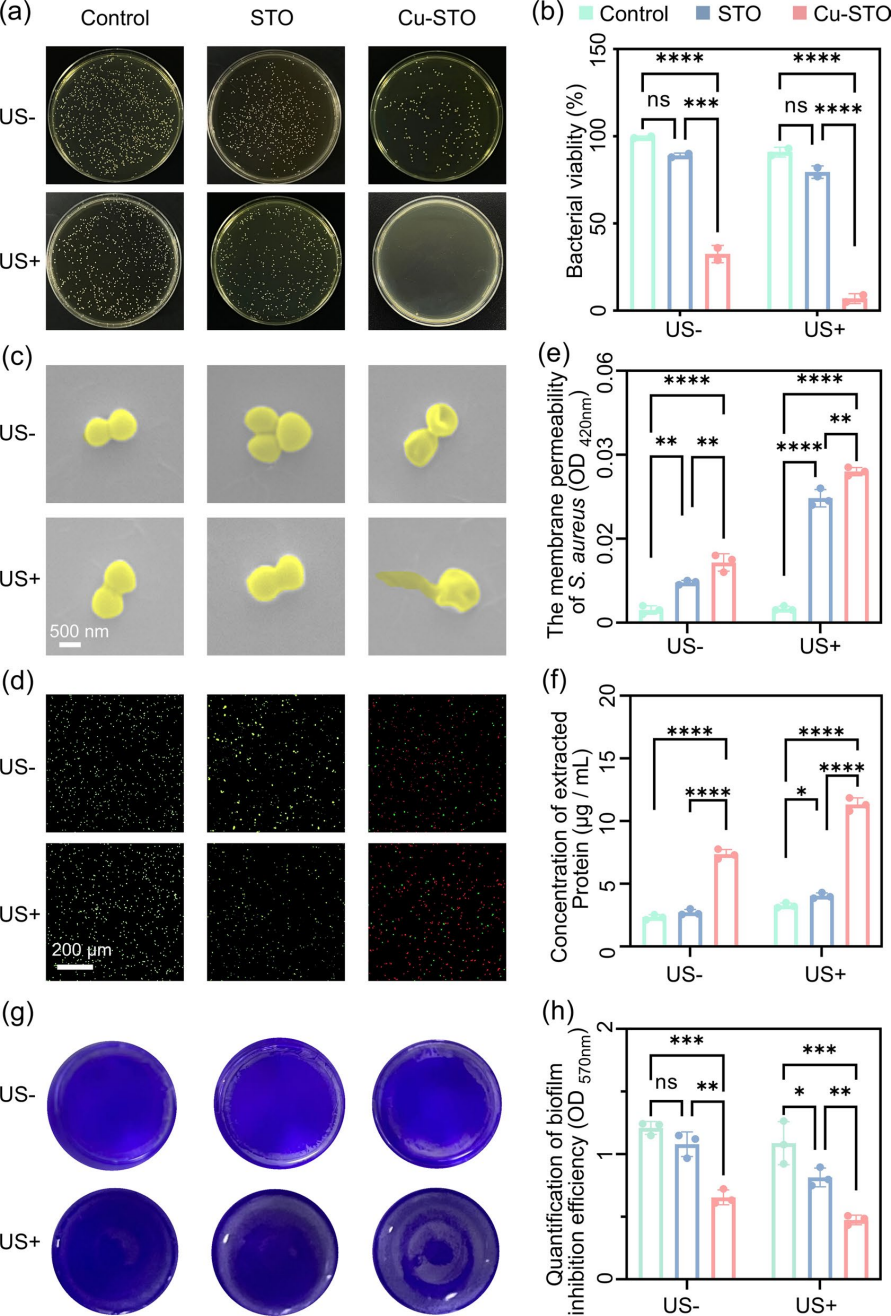
In vitro antibacterial performance of Cu-STO NSs against *S. aureus*. (**a**) Photographs and (**b**) quantitative CFU count of *S. aureus* colonies after various treatments with/without US. (**c**) SEM images and (**d)** live/dead staining (green: live; red: dead) of *S. aureus*. (**e**) Membrane permeability assessed by ONPG hydrolysis and (**f**) quantitative analysis of protein leakage. **(g**) Crystal violet-stained biofilms and (**h**) corresponding quantitative biofilm inhibition. Data are presented as mean values ± SD (n = 3 for each group). *p < 0.05, **p < 0.01, ***p < 0.001, ****p < 0.0001

Subsequently, the antibacterial efficacy of Cu-STO NSs (100 µg mL^−1^) against *S. aureus* was evaluated with and without US stimulation. As shown in Fig. 4a, neither saline nor STO showed significant antibacterial effects compared to the control. Cu-STO without US exhibited moderate antibacterial effects (67.5%), likely due to the intrinsic antimicrobial properties of copper ions. Notably, US-activated Cu-STO achieved near-complete bacterial eradication (99.3%; Fig. 4b). Furthermore, given the clinical prevalence of antibiotic-resistant strains, we evaluated the system against methicillin-resistant *S. aureus* (MRSA). As shown in Fig. S7, the Cu-STO (US+) treatment remained highly effective, achieving a > 99% reduction in MRSA viability, demonstrating that its mechanism of action is effective against drug-resistant bacteria.

Membrane integrity and bacterial morphology were then examined using SEM (Fig. 4c). Bacteria treated with Cu-STO both with and without US showed clear membrane deformation compared to the control and STO groups. Cu-STO (US+) treatment induced severe membrane shrinkage, indicating effective bacterial inactivation and correlating with its high antibacterial efficacy. Similarly, Live/Dead staining revealed extensive bacterial damage and intense red fluorescence (indicating dead cells) after Cu-STO (US+) treatment (Fig. 4d), confirming widespread bacterial eradication. Besides, membrane permeability was assessed using o-nitrophenyl β-D-galactopyranoside (ONPG) hydrolysis (Fig. 4e). Furthermore, Cu-STO (US+) induced significantly greater protein leakage from *S. aureus* than Cu-STO alone, substantially exceeding levels in the control or STO groups (Fig. 4f), confirming extensive cellular damage. The generated ROS compromise bacterial cell wall and membrane integrity, potentially impairing proton transport channels [38]. This dysfunction may facilitate substantial Cu^2+^ influx into the cytoplasm (Fig. S8), thereby inducing copper-induced metabolic toxicity.

Biofilm formation at the infection site can lead to antibiotic resistance, cause persistent infections and hinder bone regeneration and healing in severe cases. We therefore evaluated the biofilm-inhibiting properties of various samples in nutrient-rich media. After culturing *S. aureus* on different surfaces for 24 h, biofilm formation was semi-quantitatively assessed using crystal violet staining (Fig. 4g). Biofilms formed on all surfaces except Cu-STO with US irradiation. Optical density measurements further confirmed biofilm formation across the surfaces (Fig. 4h). These results indicate that Cu-STO (US+) treatment was efficient to prevent biofilm formation.

### Antibacterial mechanism of Cu-STO NSs

**Fig. 5:**
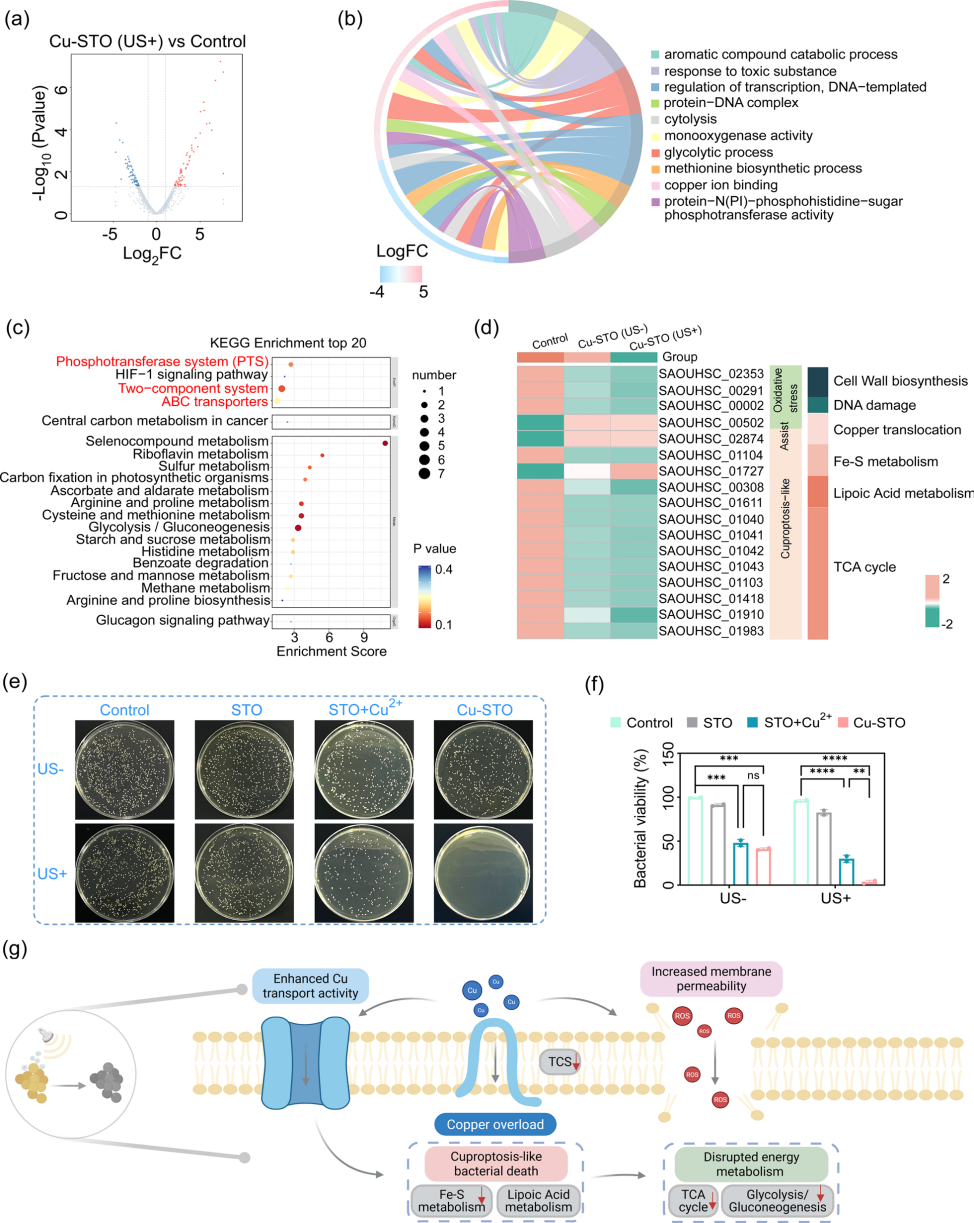
Antibacterial mechanism of Cu-STO NSs. (**a**) Volcano plot for the distribution of DEGs between Control and Cu-STO (US +). (**b**) Circular chart of GO enrichment analysis and (**c**) Bubble chart of KEGG enrichment analysis between Control and Cu-STO (US +). (**d**) Heatmap showing expression profiles of selected functional genes. (**e**) Representative images and (**f**) quantitative CFU of *S. aureus* after treatment with Control (PBS), STO, STO + Cu^2+^, Cu-STO groups. (**g**) Schematic illustration of the antibacterial mechanism via piezo-augmented copper-induced bacterial death. Data are presented as mean values ± SD (n = 3 for each group). **p < 0.01, ***p < 0.001, ****p < 0.0001

To gain deeper mechanistic insights in piezo-augmented copper-induced bacterial death, whole-transcriptome analysis of *S. aureus* was conducted across three experimental groups: Control (untreated), Cu-STO, and Cu-STO (US+). Volcano plots illustrated the distribution of differentially expressed genes (DEGs) between comparative groups (Fig. 5a and S9). Key pathway enrichment analysis using KEGG (Fig. 5b) revealed significant enrichment in the Cu-STO (US+) group for pathways associated with metal ion transport (e.g., ABC transporters), sulfur metabolism (potentially involving copper chelation), and oxidative stress response, comparing to Control group. Concurrently, Gene Ontology (GO) enrichment analysis (Fig. 5c) indicated effective suppression of both energy metabolism processes—particularly glycolysis—and essential biosynthesis pathways such as methionine synthesis, which are directly linked to the observed bactericidal effect. Heatmap analysis of specific functional genes (Fig. 5d) revealed pronounced differential expression of bacterial cuproptosis-related genes and oxidative stress in the Cu-STO (US+) group.

To further validate the synergy between piezocatalysis and Cu^2+^, the antibacterial effect of physically mixed STO and Cu^2+^ was evaluated using CFU assays (Fig. 5e, f). It is reported that minimal bacterial inhibitory concentration of Cu ions is 5 ppm [16]. The released Cu^2+^concentration was measured to be as low as 0.78 ppm (Fig. S10a). Results confirmed that Cu^2+^ supplementation at 0.78 ppm significantly enhanced antibacterial activity compared to STO alone. However, the Cu-STO heterojunction exhibited superior antibacterial efficacy under US over the physical mixture (STO + Cu^2+^), highlighting the critical role of the integrated piezocatalytic process in synergistically driving copper-induced bacterial death.

Moreover, the ROS generated by Cu-STO under US directly or indirectly inhibit the tricarboxylic acid (TCA) cycle (Fig. 5d), impairing ATP synthesis. This robust ROS production concurrently induces severe oxidative stress, damaging amino acids and enzymes essential for bacterial metabolism and biosynthesis (Fig. 5b). Critically, the US-driven piezocatalytic process increases bacterial membrane permeability (as shown in Fig. 4e-f). The antibacterial efficacy of the Cu-STO (US +) group was substantially higher than that of a STO + Cu^2+^ (US +) control group, where STO nanosheets were physically mixed with an equivalent concentration of free Cu^2+^ ions (0.78 ppm), highlighting a synergistic effect beyond simple additive mechanisms. To further quantify the individual contributions, inhibitor studies were performed. The bactericidal effect of Cu-STO (US+) was significantly attenuated in the presence of specific ROS scavengers (Vitamin C, VC) or a copper chelator (Bathocuproine disulfonate, BCS) (Fig. S11), confirming that both piezocatalytic ROS generation and bioavailable copper are indispensable components of the synergistic mechanism. This facilitated substantial intracellular overload of copper ions released from the nanosheets (Fig. S8). Transcriptomic analysis corroborates this, showing upregulation of metal ion transport pathways (Fig. 5b). The accumulated intracellular copper, particularly in the Cu^+^ state, not only catalyzes additional ROS via Fenton-like reactions but also disrupts metabolic homeostasis by oxidizing vital cellular components (e.g., lipids, proteins) [39]. This synergistic cascade of membrane damage, copper overload, and exacerbated oxidative stress leads to catastrophic metabolic dysregulation, ultimately triggering piezo-augmented copper-induced bacterial death (Fig. 5g). This integrated mechanism explains the superior efficacy of Cu-STO (US+), which achieves potent bacterial eradication even at a low released copper concentration (0.78 ppm), thereby broadening the therapeutic window of copper-induced bacterial death therapy.

### In vitro evaluation of osteogenic differentiation ability of Cu-STO NSs

**Fig. 6:**
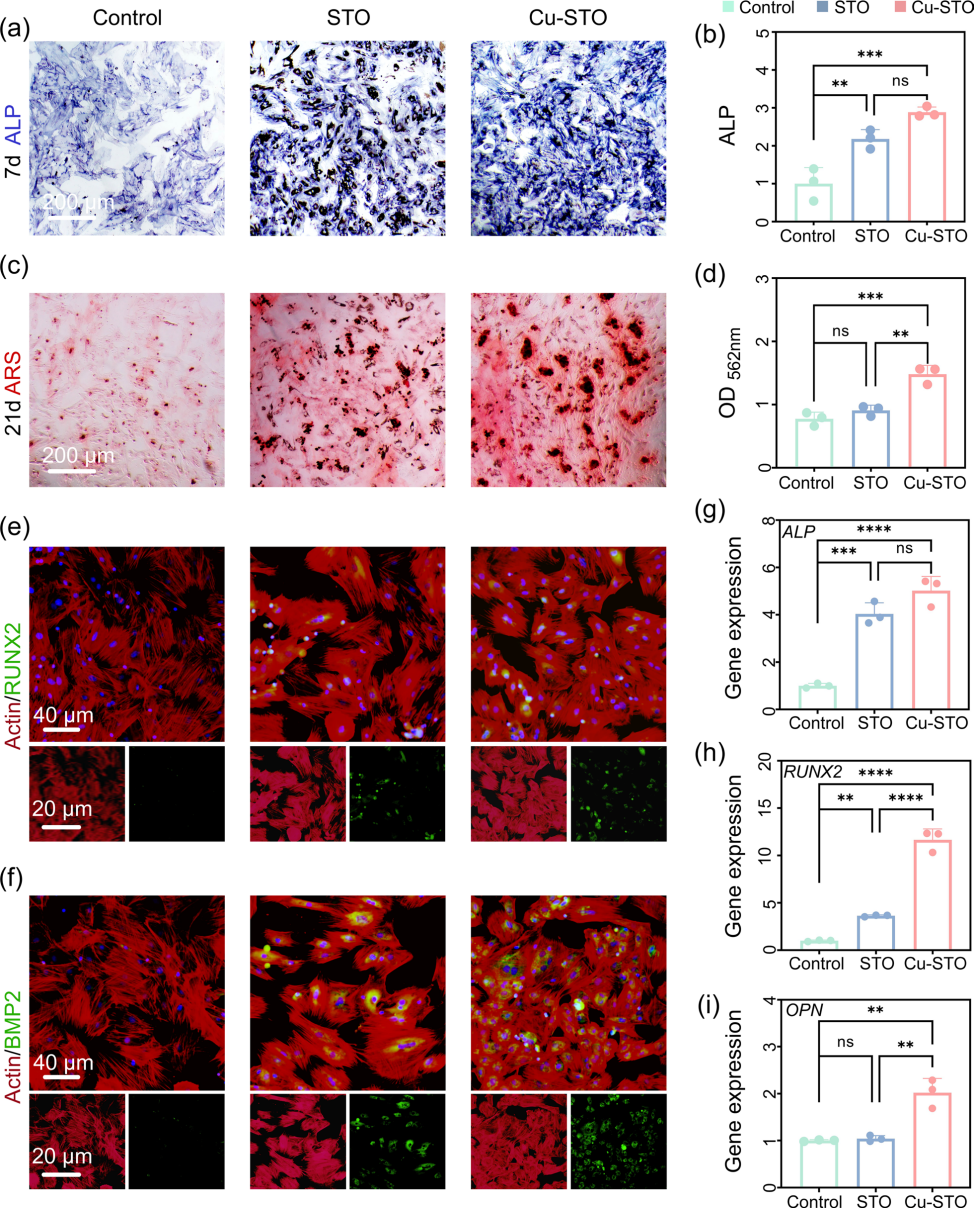
In vitro evaluation of osteogenic differentiation of BMSCs. (**a**) ALP staining and (**b**) corresponding semi-quantitative analysis of BMSCs after 7 days of culture. (**c**) ARS staining and (**d**) quantitative analysis of matrix mineralization after 21 days. Immunofluorescence staining of (**e**) RUNX2 and (**f**) BMP2 in BMSCs cultured for 14 days. (**g–i**) Relative mRNA expression levels of osteogenesis-related genes (ALP, RUNX2, OPN) in BMSCs on day 14. Data are presented as mean values ± SD (n = 3 for each group). **p < 0.01, ***p < 0.001, ****p < 0.0001

The osteogenic potential of Cu-STO NSs was then evaluated. Alkaline phosphatase (ALP) staining on day 7 (Fig. 6a, b) showed significantly higher ALP activity in the Cu-STO group versus Control and STO groups, indicating enhanced early osteogenic differentiation. Similarly, Alizarin Red S (ARS) staining on day 21 (Fig. 6c) demonstrated greater calcium deposition reflecting enhanced mineralized nodule formation in the Cu-STO group. Quantitative ARS absorbance analysis further confirmed that the Cu-STO group achieved the greatest mineralization extent (Fig. 6d). Immunofluorescence analysis demonstrated that both RUNX2 and BMP2 were significantly upregulated in BMSCs treated with STO and Cu-STO NSs. The Cu-STO group, in particular, exhibited pronounced nuclear accumulation of RUNX2 and notably intensified BMP2 signals compared to the Control after 14 days of culture (Fig. 6e, f). This enhanced protein expression was further supported at the transcriptional level, with significant upregulation of ALP, RUNX2, and BMP2 genes (Fig. 6g–i). It is reported that Cu ions upregulate the expression of Hif-1α in BMSCs by activating the Erk1/2 signaling pathway, leading to the secretion of VEGF and BMP2 proteins, thereby promoting bone formation [40]. Additionally, Sr ions in STO have been reported to not only promote osteogenesis but also inhibit bone resorption [41]. Therefore, the potent osteogenic potential Cu-STO NSs was probably attributed to the synergistic effect of Sr and Cu ions in promoting osteogenesis (ion release profile indicated in Fig. S10). Collectively, these findings exhibited Cu-STO NSs’ notable influence in facilitating osteogenic differentiation, which is beneficial for post-infection bone tissue reconstruction.

### In vivo treatment of osteomyelitis

**Fig. 7:**
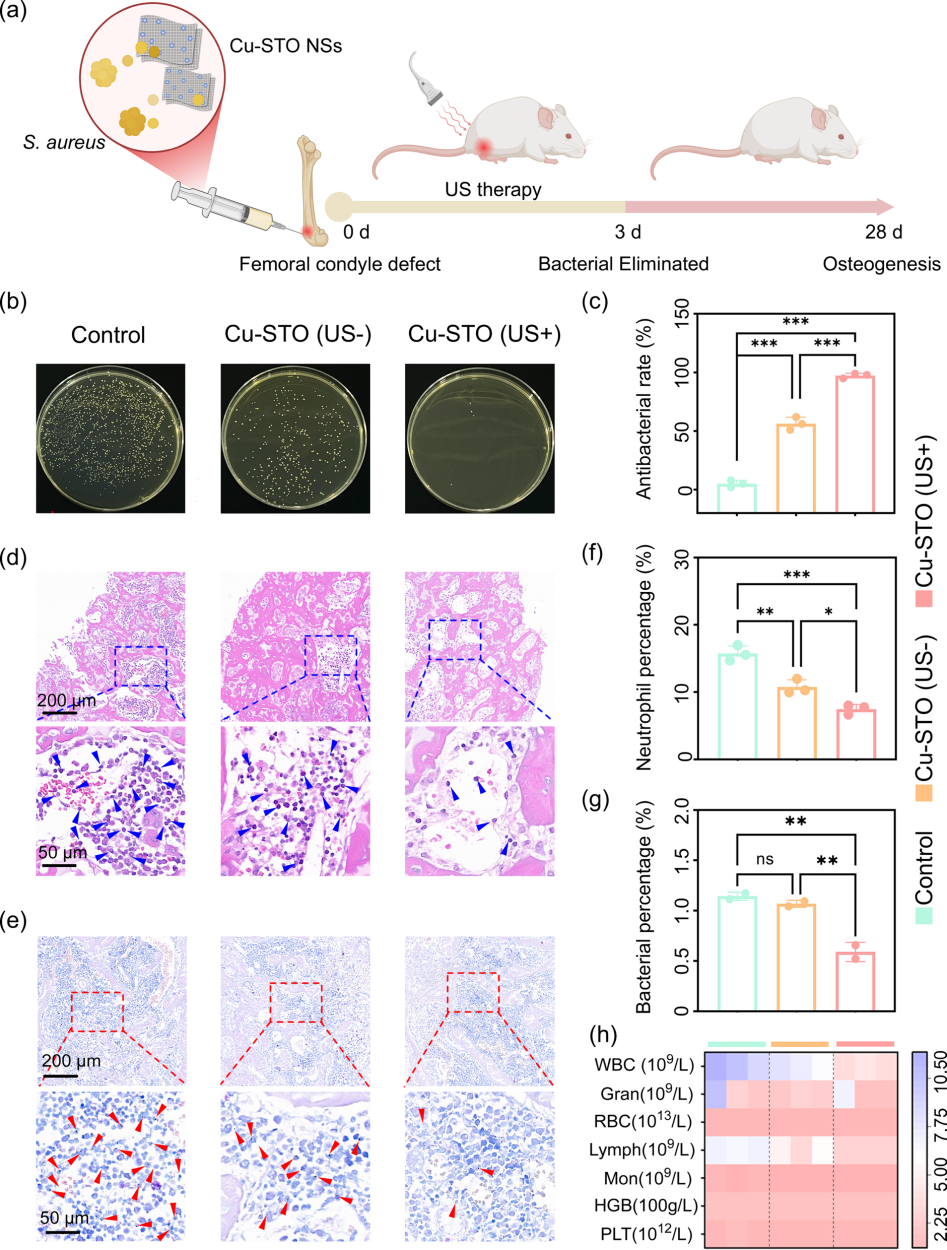
In vivo antibacterial activity of Cu-STO NSs in a rat osteomyelitis model. (**a**) Schematic illustration of the experimental timeline. (**b**) Representative images and (**c**) antibacterial rate against *S. aureus* CFU colonies harvested from infected bone tissue. (**d**) H&E and (**e**) Giemsa staining of bone sections on day 3. Semi-quantitative assessment of (**f**) neutrophil infiltration and (**g**) bacterial burden from histology. (**h**) CBC analysis on day 3. Data are presented as mean values ± SD (n = 3 for each group). *p < 0.05, **p < 0.01, ***p < 0.001

An osteomyelitis model was established in Sprague–Dawley rats via intrafemoral injection of *S. aureus* suspension (Fig. 7a). Rats were divided into three treatment groups: Control (saline), Cu-STO, and Cu-STO (US+), with the latter receiving daily US irradiation (1.5 W cm^−2^, 50% duty cycle, 1 MHz, 10 min). Femoral samples were collected at 3 days and 4 weeks post-treatment for bacteriological and histological analysis.

Quantitative culture results demonstrated a potent antibacterial effect, with the Cu-STO (US+) group achieving > 99% reduction in bacterial load compared to both control groups, confirming its superior in vivo antibacterial efficacy (Fig. 7b, c). As indicated in hematoxylin and eosin (H&E) and Giemsa staining (Fig. 7d, e), both Control and Cu-STO groups showed significant inflammatory cell infiltration and persistent bacterial colonization. Semi-quantitative scoring confirmed markedly reduced neutrophil infiltration (Fig. 7f) and bacterial burden (Fig. 7g) in the Cu-STO (US+) group, demonstrating effective infection and inflammation control. Furthermore, complete blood counts (CBC) analysis of cardiac blood samples showed significantly lower white blood cell (WBC) counts and differentials (lymphocytes, monocytes, neutrophils) in the Cu-STO (US+) group compared to the Control (Fig. 7h), indicating attenuated systemic inflammation.

**Fig. 8:**
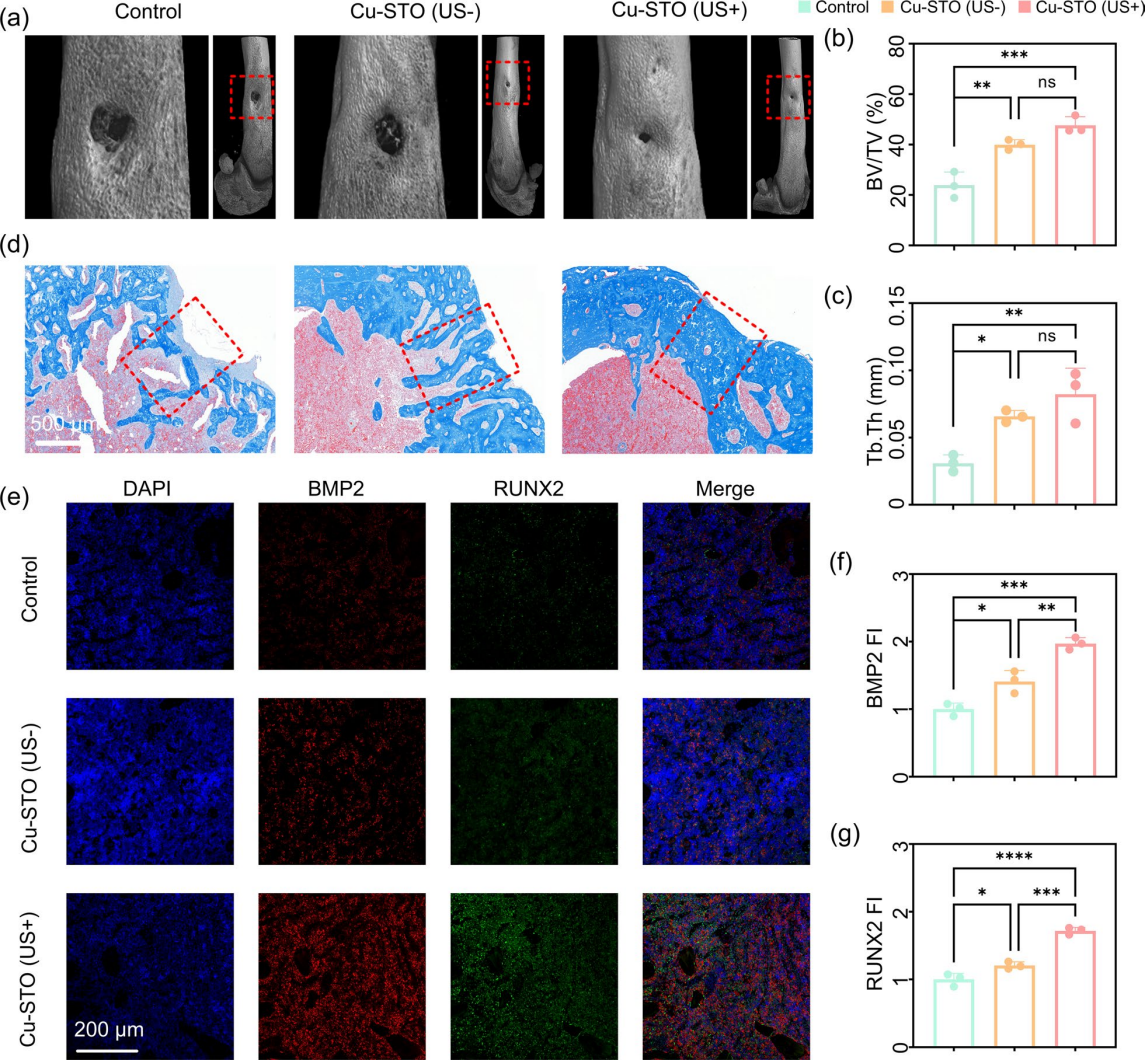
Bone regeneration performance in *S. aureus*-infected femurs. (**a**) Representative Micro-CT of infected femurs. Quantitative analysis of (b) bone volume fraction and (**c**) trabecular thickness. (**d**) Masson’s trichrome staining of bone sections. (**e**) Immunofluorescence staining for BMP2 and RUNX2, with corresponding quantitative analysis of (**f**) BMP2 and (**g**) RUNX2 expression. Data are presented as mean values ± SD (n = 3 for each group). *p < 0.05, **p < 0.01, ***p < 0.001 compared with the control

Micro-CT analysis of femurs harvested at 28 days post-treatment revealed extensive peri-defect bone loss in control animals due to infection-driven osteolysis (Fig. 8a). While the Cu-STO group exhibited moderate osteogenic activity, the Cu-STO (US+) group showed the most substantial defect repair and new bone formation. Quantitative analysis confirmed these observations, with the Cu-STO (US+) group exhibiting 1.9-fold higher bone volume fraction (BV/TV) and 2.7-fold greater trabecular thickness (Tb. Th) than the control group (Fig. 8b, c). Masson’s trichrome staining further demonstrated enhanced osteoid organization and thickness in the Cu-STO (US+) group (Fig. 8d). Immunohistochemical analysis at 4 weeks post-surgery revealed markedly elevated expression of osteogenic markers BMP2 and RUNX2 in the Cu-STO (US+) group (Fig. 8e–f), corroborating its pro-osteogenic capacity. Collectively, these findings indicate that US-responsive Cu-STO piezocatalysis effectively attenuated bone infection and subsequently promoted bone regeneration.

In addition, H&E staining of major organs (heart, liver, spleen, lung, kidney) showed no pathological abnormalities (Fig. S12), indicating negligible systemic toxicity of Cu-STO NSs. These Cu-STO NSs can be selectively and instantaneously activated only at the infection site under external US stimulation, triggering in situ copper-induced bacterial death and infection elimination. The precise focusing capability of US enables spatiotemporal control over the treatment, which significantly minimizes off-target damage to healthy tissues and addresses potential biosafety concerns.

This platform presents a dual-phase therapeutic strategy for osteomyelitis. The initial, US-triggered phase delivers rapid and potent bacterial clearance via synergistic piezocatalytic ROS generation and copper-induced metabolic toxicity, effectively sterilizing the microenvironment. This step is critical, as eliminating infection is a prerequisite for successful bone healing. Subsequently, the sustained release of bioactive Sr^2+^ and Cu^2+^ ions from the same implanted material fosters a pro-osteogenic niche, actively promoting bone regeneration as demonstrated in vitro and in vivo. This sequential coupling of infection eradication and healing stimulation within a single, multifunctional system addresses the core challenges of osteomyelitis therapy.

Regarding the pathophysiological microenvironment of osteomyelitis (hypoxia, mild acidosis), while hypoxia may constrain some oxygen-dependent ROS pathways, the acidic pH can conversely enhance the efficiency of Fenton-like reactions involving the Cu^+^/Cu^2+^ redox cycle [11]. Moreover, piezocatalysis can generate ROS from water, offering a potentially oxygen-independent route [42]. Our measurements confirmed robust intracellular ROS induction in bacteria by the Cu-STO (US+) treatment (Fig. S13).

Concerning translational safety and feasibility, the ultrasound parameters used are within clinically established safe limits. The spatiotemporally controlled, ‘on-demand’ activation confines the therapeutic action primarily to the US-targeted site, minimizing off-target effects. Crucially, the system achieves high efficacy at a very low released copper ion concentration (~ 0.78 ppm, Fig. S10a), which is significantly below the cytotoxic threshold for mammalian cells, as supported by in vitro cytocompatibility (Fig. S6) and in vivo organ histology (Fig. S12). Future studies employing chronic infection models and testing against a broader panel of resistant pathogens will further validate the platform’s potential for clinical application.

## Conclusion

In summary, we developed US-activatable Cu-STO heterojunction sub-nanosheets for synergistic treatment of osteomyelitis. The heterojunction engineered with OVs is critical for augmenting piezocatalytic performance through improved charge separation and reduced energy barriers, which effectively induces efficient bacterial copper-induced metabolic toxicity under US irradiation via membrane perturbation and copper overload. The released copper concentration can be as low as 0.78 ppm, which significant broadens the therapeutic window of copper-induced bacterial death therapy. Concurrently, the co-release of Cu^2+^ and Sr^2+^ ions promoted the osteogenic differentiation of BMSCs. Consequently, this nanosystem demonstrates robust in vivo efficacy in treating *S. aureus*-induced osteomyelitis, enabling pathogen eradication and bone regeneration. This work establishes a non-invasive, US-controlled nano-piezocatalysis platform for precise spatiotemporal regulation of the cuproptotic process that simultaneously addressing the critical challenges of infection control and bone repair in regenerative medicine.

## Supplementary Information

Supplementary Material

## Data Availability

The data that support the findings of this study are available from the corresponding author upon reasonable request.
