# Supplementary material for: Leveraging piezo-augmented copper-induced bacterial death of sub-1 nm CuO-SrTiO3-x heterojunction nanosheets for osteomyelitis eradication
Source: J Nanobiotechnology. 2026 Apr 15;24:464. doi: 10.1186/s12951-026-04380-8 (PMC13200504; doi:10.1186/s12951-026-04380-8)
Supplement: Supplementary file 1 — Supplementary Material [file 12951_2026_4380_MOESM2_ESM.docx]

**Supporting Information for**

**Leveraging Piezo-Augmented Copper-induced Bacterial Death of Sub-1 nm CuO-SrTiO_3-x_ Heterojunction Nanosheets for Osteomyelitis Eradication**

Xueqing Wang^1^, Kai Li^1^*, Wenyan Xu^1^, Danyang Wang^1^, Ying Wang^1^, Junkun Feng^1^, Yi Chen^1^, Xiaoyi Liu^1^, Zishan Xu^1^, Xiaojia Liu^1^, Shaohua Ge^1^, Hong Liu^2^, Jianhua Li^1^*

1. *Department of Biomaterials, School and Hospital of Stomatology, Cheeloo College of Medicine, Shandong University & Shandong Key Laboratory of Oral Diseases & Shandong Engineering Research Center of Dental Materials and Oral Tissue Regeneration & Shandong Provincial Clinical Research Center for Oral Diseases, Jinan 250012, China*
2. *State Key Laboratory of Crystal Materials, Shandong University, Jinan, Shandong 250100, China*

E-mail: [jianhua.li@sdu.edu.cn](mailto:jianhua.li@sdu.edu.cn)


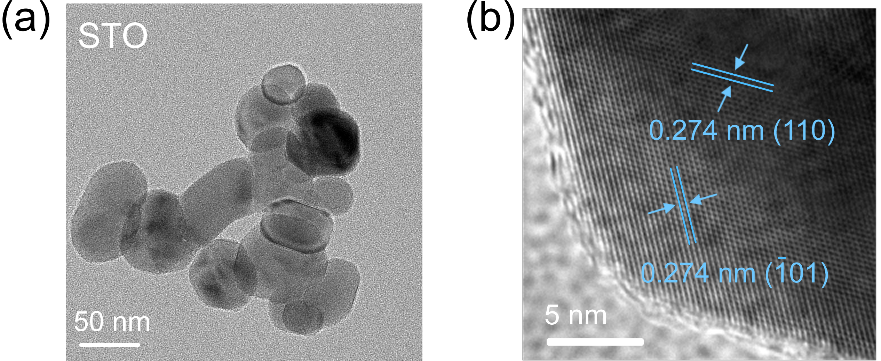


**Fig. S1.** (a) Bright-field image and (b) magnified bright-field image of STO NSs.


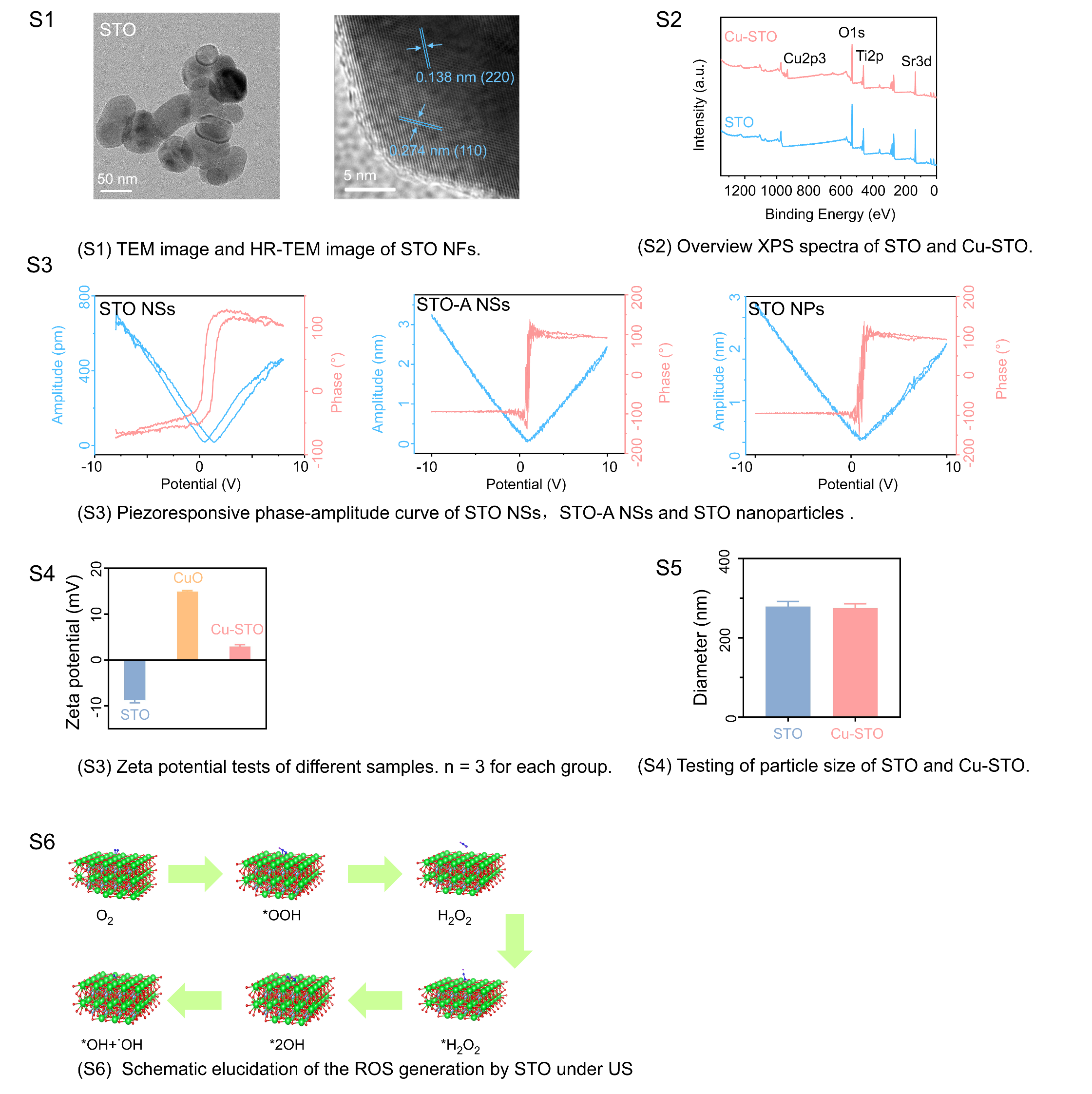


**Fig. S2.** Zeta potential measurements of STO NSs, CuO NPs and Cu-STO NSs.


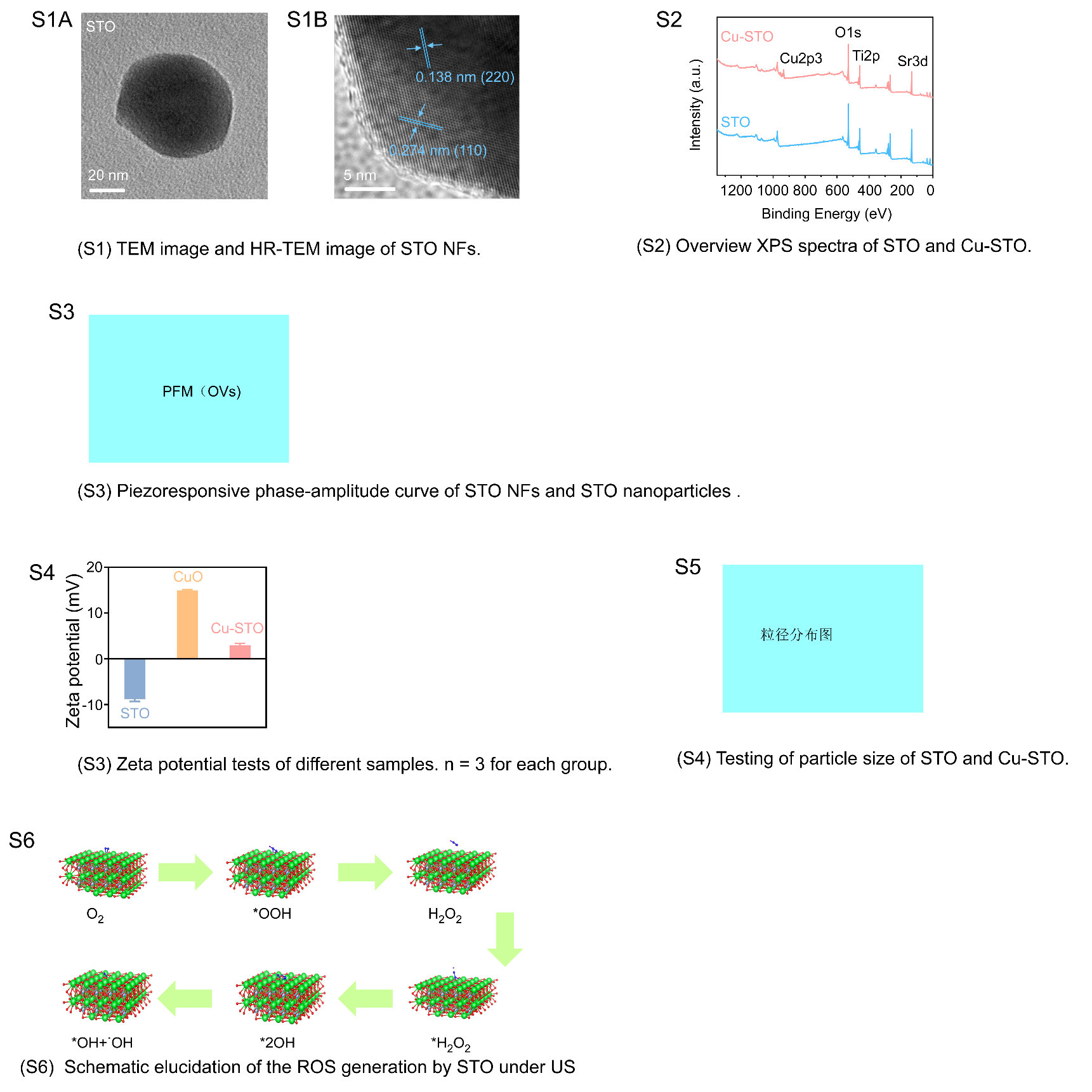


**Fig. S3.** Survey XPS spectra of STO and Cu-STO NSs.


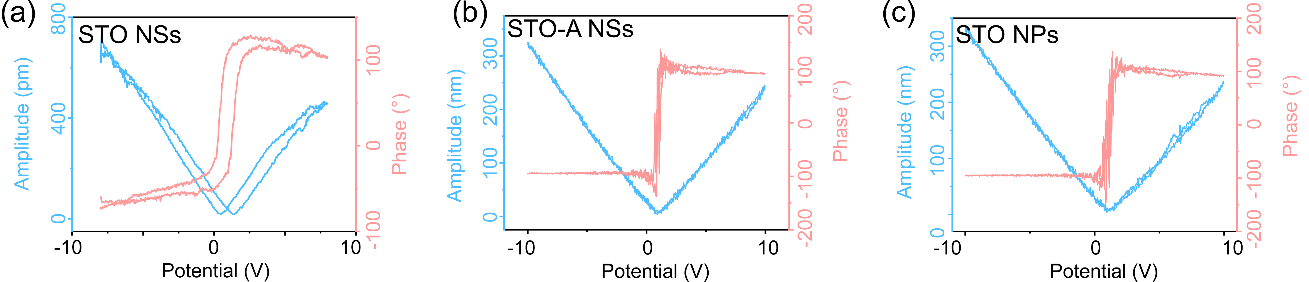


**Fig. S4.** Piezoresponsive phase-amplitude curve of (a) STO NSs, (b) STO-A NSs and (c) STO NPs.


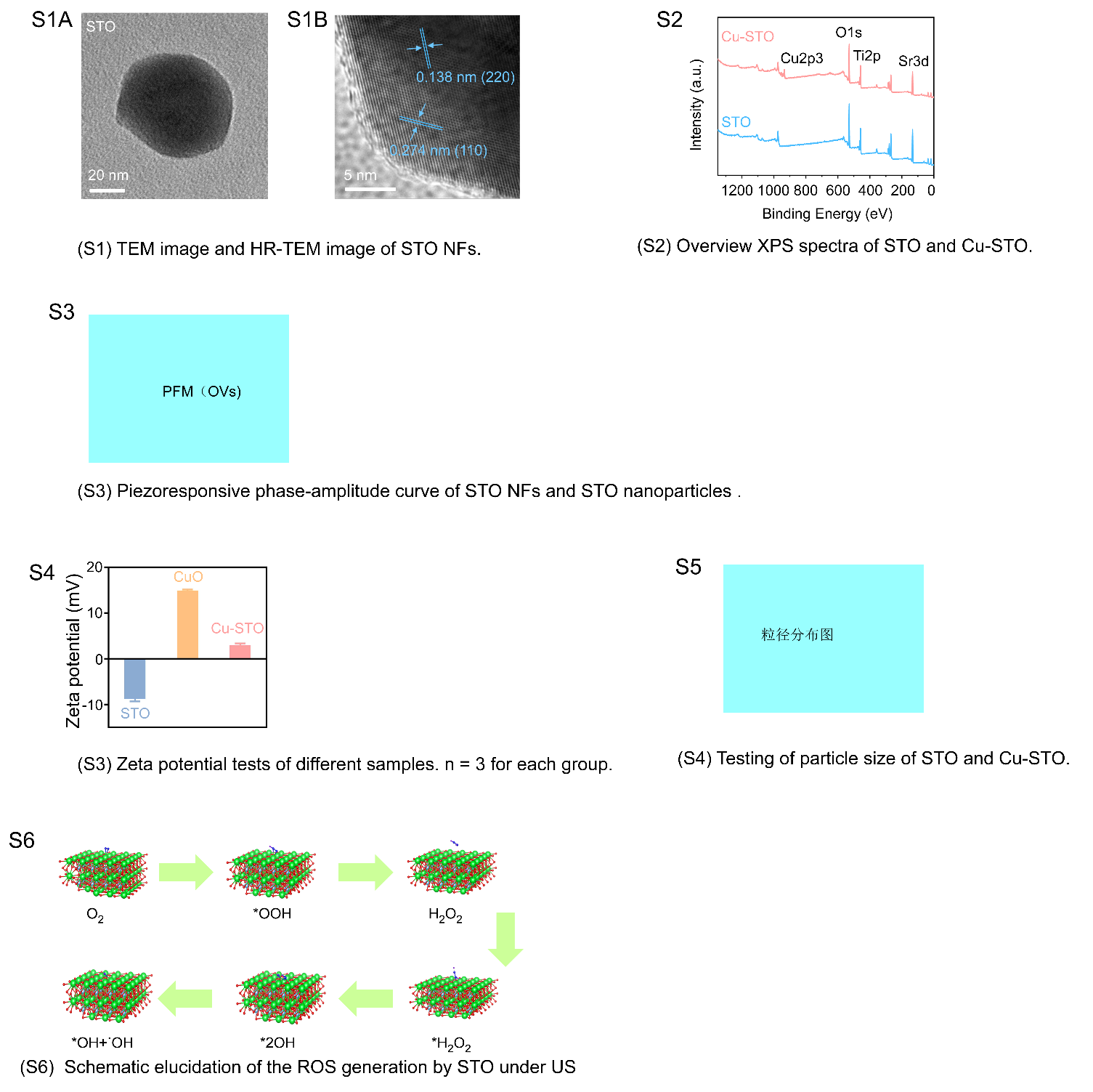


**Fig. S5.** Key intermediate conformations of STO in tandem reactions for •O_2_^-^ to •OH conversion.


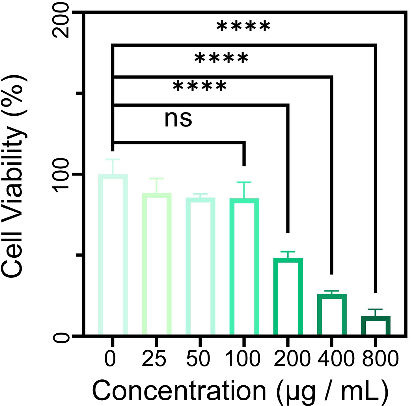


**Fig. S6. Viability of MC3T3-E1 cells evaluated by CCK-8 assay after treatment with samples at concentrations ranging from 0 to 800 μg mL^-1^. Data are presented as mean values ± SD (n = 3 for each group). ****p < 0.0001.**


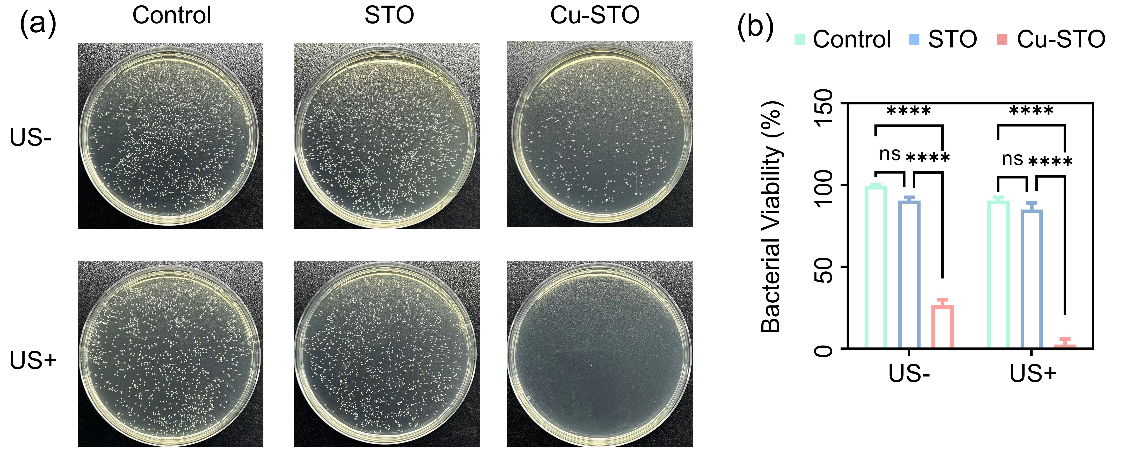


**Fig. S7. *In vitro* antibacterial performance of Cu-STO NSs against MRSA. (a) Photographs and (b) quantitative CFU count of MRSA colonies after various treatments with/without US. Data are presented as mean values ± SD (n = 3 for each group). ****p < 0.0001.**


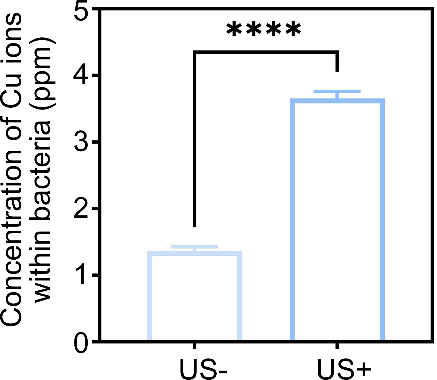


**Fig. S8.** Intracellular Cu ion concentration in S. aureus before and following Cu-STO (US+) treatment. Data are presented as mean values ± SD (n = 3 for each group). ****p < 0.0001 compared with the control.


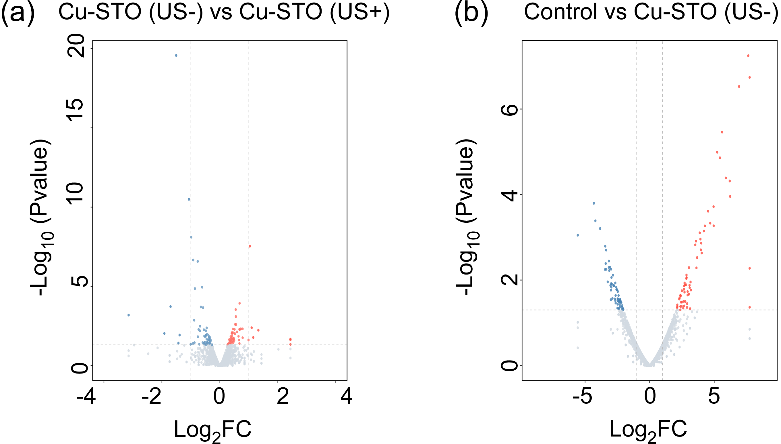


**Fig. S9.** Volcano plot showing the distribution of differentially expressed genes (DGEs).


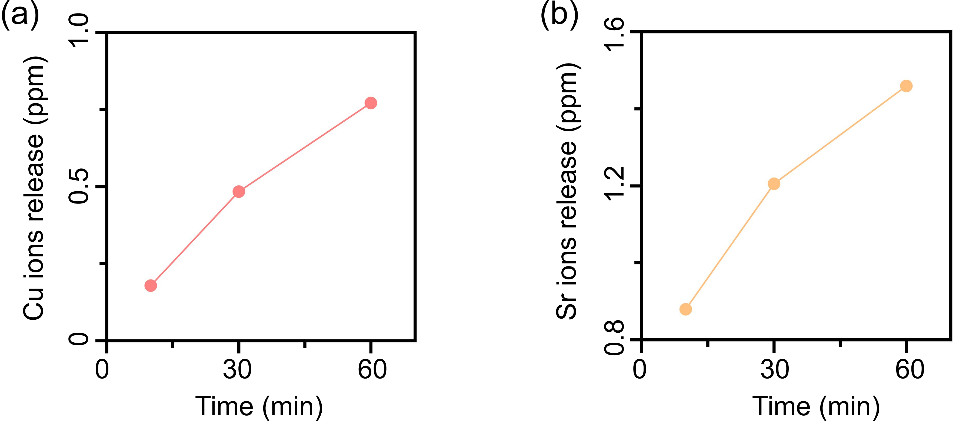


**Fig. S10.** Time-dependent release profiles of (a) Cu and (b) Sr ions from Cu-STO NSs incubated in 0.9% NaCl solution at 37 °C. Ion concentrations were measured by ICP-OES after 10, 30, and 60 minutes of incubation.


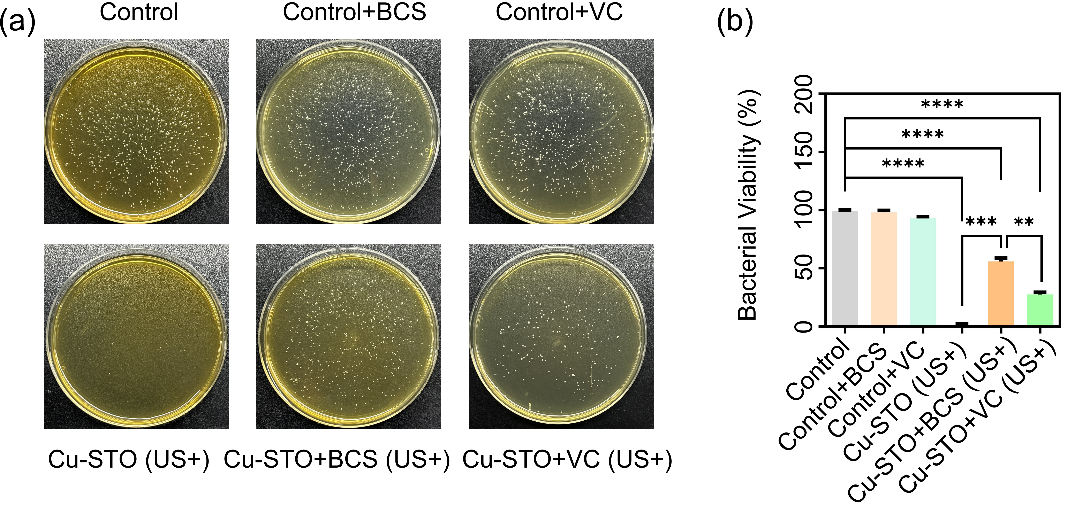


**Fig. S11.** Inhibition assays evaluating the contribution of ROS and copper ions to the antibacterial activity of Cu-STO (US+). (a) Representative photographs and (b) quantitative colony-forming unit (CFU) counts of *S. aureus* after various treatments: Control (PBS), Control with the copper chelator bathocuproine disulfonate (BCS), Control with the ROS scavenger ascorbic acid (VC), Cu-STO with ultrasound (US) activation, Cu-STO (US+) with BCS, and Cu-STO (US+) with VC. The significant reduction in bactericidal efficacy upon addition of either BCS or VC confirms that both copper ions and reactive oxygen species are essential for the synergistic antibacterial effect. Data are presented as mean values ± SD (n = 3 for each group). **p < 0.01, ***p < 0.001, ****p < 0.0001.


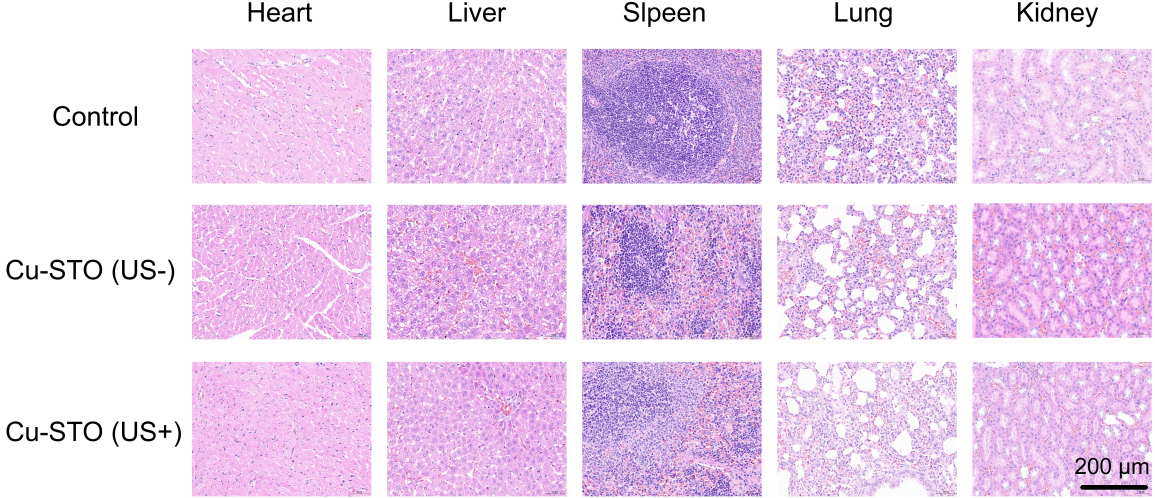


**Fig. S12.** H&E staining of major organs (heart, liver, spleen, lung, and kidney) from each treatment group. Representative images from three independent biological replicates are shown.


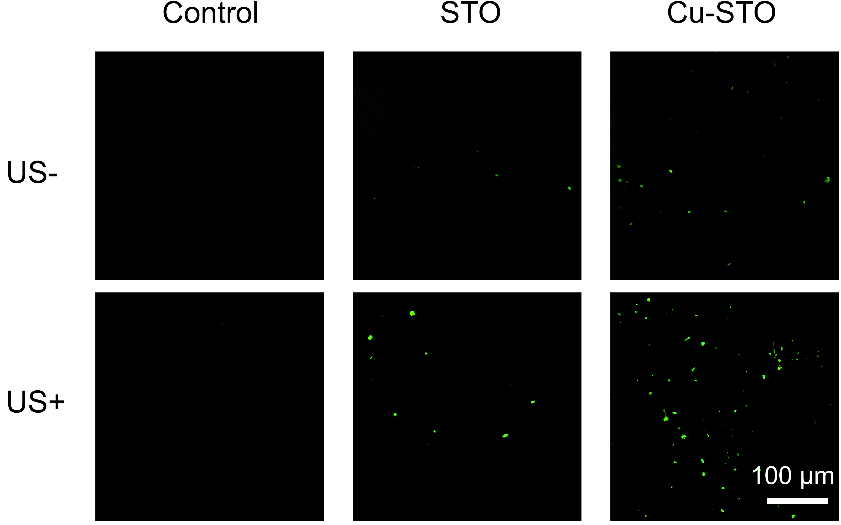


**Fig. S13.** Intracellular reactive oxygen species (ROS) generation in *S. aureus* triggered by different treatments. ROS levels were measured using the fluorescent probe DCFH-DA. Treatments include: Control (PBS), STO nanosheets alone (STO), STO (US+), copper-decorated STO nanosheets without US (Cu-STO (US-)), and copper-decorated STO nanosheets with US activation (Cu-STO (US+)). The Cu-STO (US+) group induced the most pronounced ROS burst, correlating with its superior antibacterial efficacy.

**Table S1.** The primers for the target genes.

| Gene | Forward primer sequence (5’–3’) | Forward primer sequence (5’–3’) | Annealing temperature(°C) | | Product size(bp) |
| --- | --- | --- | --- | --- | --- |
| ALP | ACAACACCAACGCTCAGGTC | GTGACCTCGTTCCCCTGAGT | | 60 | 142 |
| OPN | GGTGATAGCTTGGCTTACGGA | TGGCATCGGGATACTGTTCA | | 60 | 76 |
| Runx-2 | ACGAATGCACTATCCAGCCA | GCAGGTACGTGTGGTAGTGA | | 60 | 98 |
| GAPDH | CTGGAGAAACCTGCCAAGTATG | GGTGGAAGAATGGGAGTTGCT | | 60 | 138 |
